# Whole-Exome Sequencing-Based Mutational Profiling of Hepatitis B Virus-Related Early-Stage Hepatocellular Carcinoma

**DOI:** 10.1155/2017/2029315

**Published:** 2017-11-29

**Authors:** Hao Zhan, Jiahao Jiang, Qiman Sun, Aiwu Ke, Jinwu Hu, Zhiqiang Hu, Kai Zhu, Chubin Luo, Ning Ren, Jia Fan, Jian Zhou, Xiaowu Huang

**Affiliations:** ^1^Liver Cancer Institute, Key Laboratory of Carcinogenesis and Cancer Invasion, Ministry of Education, Zhongshan Hospital, Fudan University, Shanghai, China; ^2^Department of Thoracic Surgery, Zhongshan Hospital, Fudan University, Shanghai, China; ^3^Shanghai Key Laboratory of Organ Transplantation, Shanghai, China

## Abstract

**Background:**

Hepatocellular carcinoma (HCC) ranks as the third leading cause of cancer-related mortality in China with increasing incidence. This study is designed to explore early genetic changes implicated in HCC tumorigenesis and progression by whole-exome sequencing.

**Methods:**

We firstly sequenced the whole exomes of 5 paired hepatitis B virus-related early-stage HCC and peripheral blood samples, followed by gene ontological analysis and pathway analysis of the single-nucleotide variants discovered. Then, the mutations of high frequency were further confirmed by Sanger sequencing.

**Results:**

We identified a mutational signature of dominant T:A>A:T transversion in early HCC and significantly enriched pathways including ECM-receptor interaction, axon guidance, and focal adhesion and enriched biological processes containing cell adhesion, axon guidance, and regulation of pH. Eight genes, including MUC16, UNC79, USH2A, DNAH17, PTPN13, TENM4, PCLO, and PDE1C, were frequently mutated.

**Conclusions:**

This study reveals a mutational profile and a distinct mutation signature of T:A>A:T transversion in early-stage HCC with HBV infection, which will enrich our understanding of genetic characteristics of the early-stage HCC.

## 1. Introduction

Hepatocellular carcinoma (HCC) ranks as the third leading cause of cancer-related mortality in China [1]. In the meantime, its incidence tends to increase over the past decade [2]. In 2012, GLOBOCAN reported around 782,500 new cases of liver cancer [2]. Although massive advances in treatments for viral hepatitis and updated means to diagnose and treat liver tumours were achieved in the recent years, the prognosis of this malignancy remains to be dismal [3, 4]. With the incidence rate approximating the death rate, most of the HCC patients die of this lethal disease [5].

A multistep process of sequential genetic changes is a core driving force behind the development and progression of cancer [6]. In this era of novel high-throughput genome and exome sequencing technologies, it is feasible to explore cancer genomes at a high resolution and catalogue each gene mutation in a given human cancer. Recent sequencing studies of exomes and genomes in multiple HCC cohorts have identified numerous novel molecular alternations of various aetiologies, including recurrent mutations in the genes of *TP53*, *ARID2*, *ARID1A*, *CTNNB1*, *AXIN1*, *RPS6KA3*, and *IRF2* [7–10], and major pathways that are commonly altered, such as the chromatin remodelling and Wnt/beta-catenin pathways [7, 9]. However, HCC-associated somatic mutations vary extensively among individuals and even within a single tumour. Because of this dramatic diversity, no clear genetic signatures have been identified to stratify mutations, and pharmaceutical endeavours investigating new targeting strategies against HCC have encountered enormous challenges [11]. The only FDA-approved drug to treat advanced HCC, Sorafenib, merely prolongs patients' lifespans by an average of 2.8 months and is accompanied by a series of side effects [12]. Therefore, additional efforts to characterize the molecular pathogenesis of this malignancy are still imperative.

With Over 240 million people chronically infected worldwide [3], hepatitis B virus (HBV) is the most significant factor underlying the development of HCC, which accounts for more than 3/5 of all cases of liver cancer in the developing countries and a little less than 1/4 of cases in the developed countries [13]. Various risk factors for HCC may affect the mutation profile of the corresponding cancer genome [14]. In addition, targeting early steps in the development of cancer limits the genetic variety that develops over time. Based on the above considerations, we performed a whole-exome sequencing study on 5 paired early-stage HCC (eHCC) with HBV infection and peripheral blood samples. This study is designed to decipher the genetic changes involved in HCC tumorigenesis and progression and to advance the development of therapeutic strategies and early diagnosis.

## 2. Materials and Methods

### 2.1. Patients and Samples

Five paired HCC specimens and peripheral blood samples were collected at the Liver Cancer Institute of Zhongshan Hospital (Fudan University, Shanghai, China) from patients receiving curative resection, and the samples were immediately frozen at −80°C, followed by pathological examination and confirmed to be HCC by experienced pathologists. These patients received no prior therapies, such as transarterial chemoembolization or radiofrequency ablation. Zhongshan Hospital Ethics Committee approved this study, and each patient signed informed consent following institutional review board protocols.

### 2.2. Whole-Exome Capture, Sequencing, and Mutation Analysis

Using a DNeasy tissue kit (Qiagen), paired tumour/normal genomic DNA from 5 patients were extracted and randomly sonicated to generate 150–200 bp products. After end repair with 3′A overhang and NimbleGen linker adaptation, the AMPure XP bead-purified DNA fragments were subjected to LM-PCR. In accordance with the manufacturer's protocol, the prepared DNA libraries were hybridized to Agilent SureSelectXT Human All Exon kit to capture the target exome. After another round of LM-PCR, a minimum of 80-fold enrichment was confirmed by qPCR for all of the captured sequences. The exonic DNA library was then sequenced on the Illumina HiSeq2000 platform. We aligned the sequenced reads to the human reference genome (GRCh37/hg 19) from the UCSC Genome Browser by Burrows-Wheeler Aligner (BWA, version 0.6.2) software and removed the PCR duplicates by SAMtools software. To detect potential variants, we employed the Genome Analysis Toolkit (GATK) to perform consensus calling, and the discovery pipeline is detailed in Figure S1. The somatic variants from each paired sample were justified by the following criteria: (1) phred-scaled consensus scores > 20 and mapping qualities > 30; (2) variant reads ≥ 10% of the total reads; and (3) number of variant reads ≥ 5 and total reads ≥ 10. We removed common polymorphisms by comparison to dbSNP135 and the 1000 Genomes Project database (http://www.1000genomes.org), as well as synonymous mutations and variants found in normal exomes. The Integrated Genomics Viewer (IGV) software was used to examine all the candidate mutations, and the selected SNVs were confirmed by PCR-based Sanger sequencing in each tumour tissue and paired blood sample. The mutational signatures of each tumour were extracted by the application of deconstructSigs, as described before [15].

### 2.3. Gene Function Analysis

The identified genes with SNVs were further input into Database for Annotation, Visualization and Integrated Discovery (DAVID; http://david.abcc.ncifcrf.gov) v6.7 using GO to reveal the molecular function denoted in the gene profile. Simultaneously, these genes were input into the Kyoto Encyclopedia of Genes and Genomes (KEGG) to analyze the pathway associated [16]. The cut-off *P* value of 0.05 is recommended.

## 3. Results

### 3.1. Landscape of Somatic Mutations in HBV-Related eHCC

**Figure 1:**
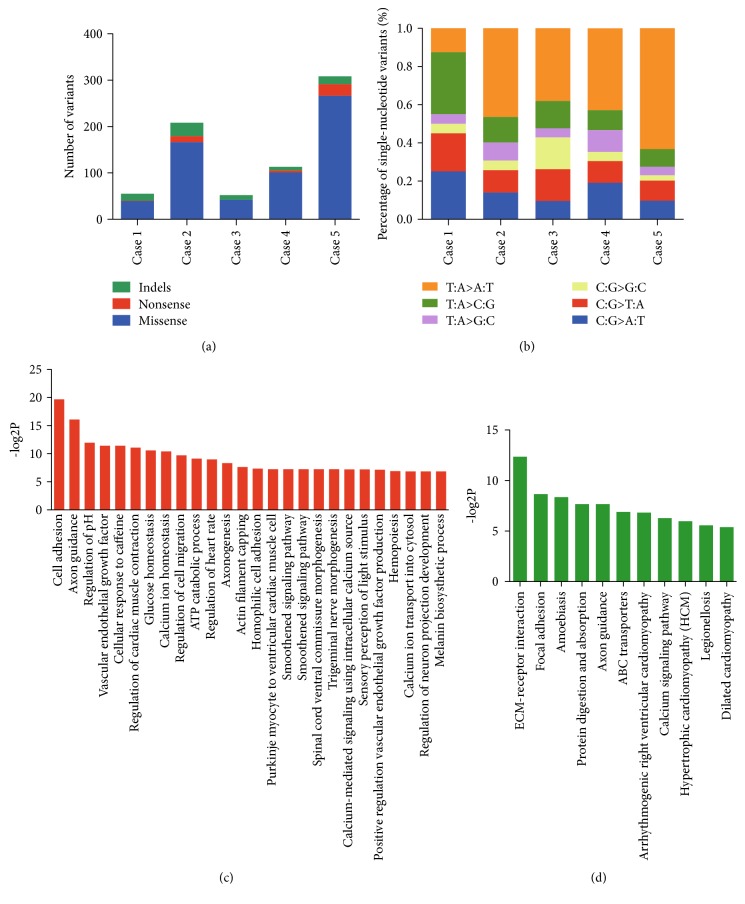
Mutational profile of HBV-related eHCC and affected biological processes and pathways. (a) Numbers of each type of variant in 5 cases. (b) Mutational spectra of single-nucleotide variants in 5 cases. (c, d) Significantly enriched biological processes (c) and pathways (d) affected by the SNVs in our HBV-related eHCC cohort.

To obtain insight into the genetic basis of HBV-related eHCC, exome sequencing was performed on paired tumour and peripheral blood DNA from 5 HCC patients (BCLC stage A, 1 tumour nodule, diameter ≤ 3 cm; Table 1). The mean sequence coverage depth of targeted exonic regions reached 80.7-fold (ranging from 68.3-fold to 92.2-fold), and the median depth was 63.4-fold (ranging from 54-fold to 73-fold). Over 95% of the targeted exome gained coverage of at least one read (Table S1). Somatic single-nucleotide variants (SNVs) and small insertions and deletions (indels) were predicted using algorithms described in Figure S1. Briefly, to remove common germline variants, we removed candidate mutations that already existed in dbSNP135 or in the 1000 Genomes Project dataset, and tumour-specific mutations were confirmed by comparing the tumour data with those of matched peripheral blood samples. On the whole, we identified 736 potential protein-altering somatic mutations within 708 genes, with 614 missense mutations, 43 nonsense mutations, and 79 small indels (Table S2 and S3); this pattern is analogous to the distribution found in solid tumours [17]. The number of somatic mutations per tumour ranged from 52 to 308 (Figure 1(a)), which was comparable to values reported in previous studies [7–9].

Notably, a predominance of T:A>A:T transversions with a strong transcriptional strand bias was identified, which affected 4 out of 5 individuals in our cohort (Figure 1(b)). In case 5, the mutation rate of this transversion reached 63.2% among all mutation types. After using the deconstructSigs method to extract the exact signatures underlying these SNVs of each tumour sample, the signature 22, as documented in Catalogue of Somatic Mutations in Cancer (http://cancer.sanger.ac.uk/cosmic/signatures), consisting of dominated T:A>A:T transversions, was found overrepresented in 4 out of 5 HBV-related eHCC and when all the SNVs were pooled together (Figure S2). Additionally, an overrepresentation of transversions over transitions (rate, 3.11) was found, in agreement with the results of previous HCC sequencing studies [9, 10].

To better understand the potential underlying mechanisms implicated in HBV-related eHCC, we performed GO (Gene Ontology) and pathway analysis based on the identified SNVs (Table S4 and S5) and found that pathways associated with extracellular matrix- (ECM-) receptor interaction (*P* = 1.91*E* − 04, false discovery rate [FDR] = 0.04), focal adhesion (*P* = 2.49*E* − 03, FDR = 0.19), and amoebiasis (*P* = 3.05*E* − 03, FDR = 0.19) were the top 3 significantly enriched pathways and that cell adhesion (*P* = 1.17*E* − 06, FDR = 0.00), axon guidance (*P* = 1.43*E* − 05, FDR = 0.01), and regulation of pH (*P* = 2.48*E* − 04, FDR = 0.13) were the top 3 significantly enriched biological processes (Figures 1(c) and 1(d)).

### 3.2. Candidate Somatic Mutations Revealed by Exome Sequencing

**Figure 2:**
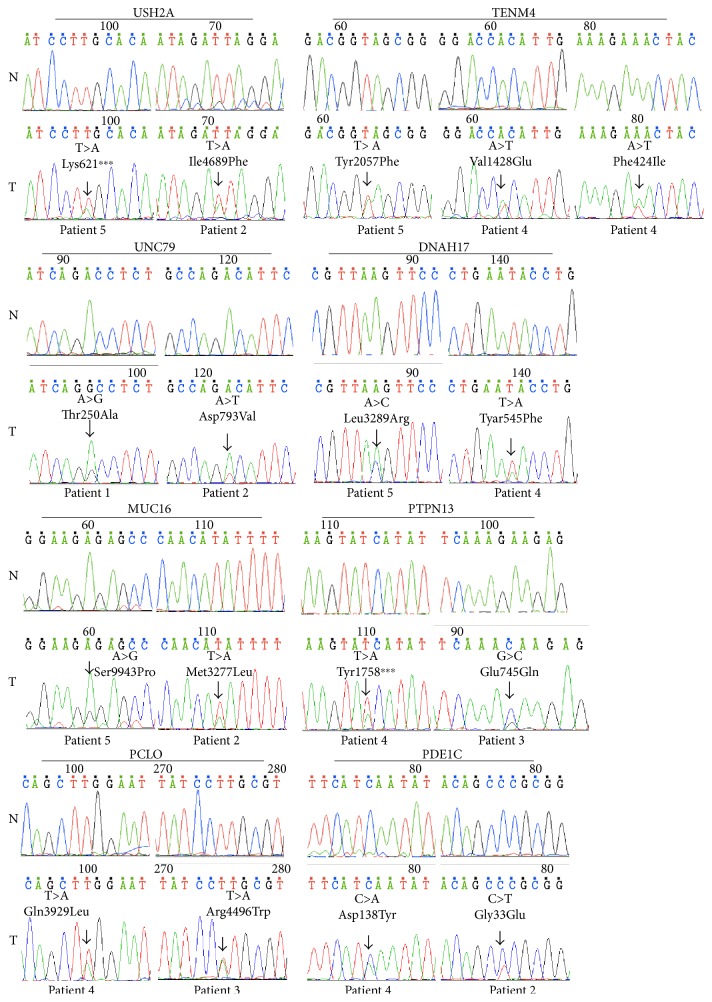
Recurrent mutations verified by Sanger sequencing. Frequent mutations in 2 cases were verified by Sanger sequencing. Each mutation was heterozygous and led to a change in the amino acid sequence of the encoded protein. None of the mutations reside in the DNA of peripheral lymphocytes. The black arrows indicate the mutation site. N: peripheral lymphocytes. T: HCC sample.

In total, 8 genes, including MUC16, UNC79, USH2A, DNAH17, PTPN13, TENM4, PCLO, and PDE1C, were found to be mutated in at least 2 HCC samples (Table 2); 17 candidate somatic mutations located on these genes were further confirmed via PCR-based Sanger sequencing (Figure 2, Table S6). Each mutation was heterozygous, leading to sequence changes of the corresponding amino acid in the encoded protein.

## 4. Discussion

Cancer is a disease of the genome [18]. Genetic alterations, which confer selective growth advantages, sequentially accumulate during tumorigenesis due to various factors and mechanisms. Although recent exome and genome sequencing studies have examined large collections of HCC cases and refined the mutational landscape, our knowledge of the driving mutations and related signaling pathways involved in the tumorigenesis and progression of this malignancy is still far from complete, which precludes the use of genetic testing to categorize patients and target therapy.

Mutational signatures differ vastly among cancers [17]. Various carcinogens may also affect somatic substitution patterns in HCCs, and the mutation spectrum identified could indicate the specific pattern of mutagenesis occurring in tumour cells [19]. Through whole-exome sequencing, Huang et al. recently identified dominant G:C>T:A and T:A>A:T transversions in relatively advanced HCCs with portal vein thrombosis [10]. Based on exome sequencing, a predominance of T:A>A:T, but not G:C>T:A, transversions in eHCC was identified. This mutation signature strongly differs from what have been observed in other solid tumours, in which C:G>T:A transitions are the dominant alterations [17]. Remarkably, the discrepancy in the mutation spectra between our study and Huang's suggests that distinct mutational signatures may arise at different stages (e.g., early and advanced stages) of a cancer. T:A->A:T transversions are also well documented in urothelial carcinoma after exposures to aristolochic acid (AA), and often, the signature 22 indicates AA exposure [20]. Though with this signature similarity, AA is not a common cause of HCC, and the dominance of the T:A>A:T transversion was repeatedly discovered in the present study without proof of AA exposure and others [10, 21], which can hardly all be attributed to unexpected AA exposure. Thus, the dominant T:A>A:T transversions could possibly be a distinctive mutational signature of HBV-related eHCCs, yet this assumption must be tested in larger samples of HBV-related eHCCs.

Using mutated genes affected by SNVs in 5 cases, we carried out GO and pathway analysis and identified ECM-receptor interaction, axon guidance, and focal adhesion as significantly mutated pathways and/or biological processes. Notably, axon guidance was identified as both significantly mutated pathway and biological process. 12 mutated genes involved in this pathway affected 4 out of 5 cases in this cohort. The axon guidance genes were originally characterized by their roles in guiding axon pathfinding to its synaptic targets, thereby forming neural connections and wiring the nervous system during embryogenesis [22]. Recent sequencing studies have revealed frequent and diverse somatic mutations in axon guidance genes in various human cancers, including gastric cancer, pancreatic cancer, and liver fluke-associated cholangiocarcinoma, highlighting their involvement in driving malignancies [23–25]. Slit and Roundabout (Robo) are both axon guidance molecules. Compelling evidence reasoned that Slit/Robo signaling inhibited E-cadherin-mediated cell adhesion and induced EMT-like phenotype in colorectal carcinoma [26]. EMT is a complex biological process by which epithelial cells shed their differentiated features (e.g., disrupted intercellular adhesion and loss of cell polarity) and instead assume a mesenchymal phenotype, including capabilities to degrade ECM, enhanced motility/invasiveness, and heightened resistance to apoptosis [27]. Accumulating observations point to its pivotal role in carcinogenesis and cancer-related invasion and metastatic dissemination [28, 29]. In addition, the ECM-receptor interaction and focal adhesion pathways were also associated with EMT. The significantly enriched pathways involved in EMT identified in our eHCC study imply that genetic alterations favouring metastatic progression are acquired at an early stage of HCC development.

Of the 8 frequent mutated genes, 7 (except *TENM4*) were annotated in the Catalogue of Somatic Mutations in Cancer (COSMIC) and also reported in recent sequencing studies of HCC [9, 30], as well as a variety of cancer types. *MUC16* and *USH2A* were mutated with over 5% frequency in documented tissue samples. Elevated expression of MUC16 has been found in breast cancer and late stage and metastatic sites of pancreatic cancer [31, 32]. Eudy et al.demonstrated that MUC16 inhibits NK cell-mediated cytotoxicity against ovarian cancer cells. Due to its involvement in ECM constitution and cellular adhesion [33], the mutation of *USH2A* could possibly facilitate the process of EMT. The nonsense mutation (Tyr1758^∗∗∗^) in *PTPN13* leads to loss of function in accord with the prior study reasoning *PTPN13* as a candidate tumour suppressor gene in HCC [34]. These observations may imply that the overlapping mutated genes may harbor driver mutations of this malignancy.

In conclusion, our work documents the mutational profile, affected pathways, a distinct T:A>A:T transversion signature, and frequent mutated genes in HBV-related eHCC, which enriched our understanding of the biology underlying the tumorigenesis of HCC.

## Figures and Tables

**Table 1:** Clinicopathologic characteristics of 5 hepatocellular carcinoma patients used for exome sequencing.

Patient ID	Gender	Age	Aetiology	Liver cirrhosis	Edmondson grade	Adjacent invasion	Vascular invasion	Lymph node	Metastasis	Tumour size (cm)	TNM stage^∗^	BCLC stage
Patient 1	Male	45	HBV	Yes	II	No	No	No	No	2.8 × 2.8	II	A
Patient 2	Male	52	HBV	Yes	II	No	No	No	No	2 × 1.8	I	A
Patient 3	Male	42	HBV	No	II	No	No	No	No	2 × 1.8	I	A
Patient 4	Male	52	HBV	No	I	No	No	No	No	2 × 1.8	I	A
Patient 5	Male	61	HBV	Yes	II	No	No	No	No	2.2 × 2.2	II	A

^∗^Tumour staging was based on the 7th edition of tumour-node-metastasis classification of the American Joint Committee on Cancer Staging Manual. HBV: hepatitis B virus; BCLC: Barcelona clinic liver cancer.

**Table 2:** Summary of frequent mutations in 8 genes verified by Sanger sequencing.

Gene	Gene ID	Chromosome^#^	Sample ID	Position	Reference base	Mutation base	Protein annotation	Mutation type	Sanger-seq verified
USH2A	NM_206933.2	chr1	Patient 5	216462732	T	A	Lys621^∗∗∗^	Nonsese	Yes
			Patient 2	215844382	T	A	Ile4689Phe	Missense	Yes

TENM4	NM_001098816.2	chr11	Patient 5	78381220	T	A	Tyr2057Phe	Missense	Yes
			Patient 4	78413375	A	T	Val1428Glu	Missense	Yes
			Patient 4	78567209	A	T	Phe424Ile	Missense	Yes

UNC79	NM_020818.3	chr14	Patient 1	94004491	A	G	Thr250Ala	Missense	Yes
			Patient 2	94053131	A	T	Asp793Val	Missense	Yes

DNAH17	NM_173628.3	chr17	Patient 5	76454758	A	C	Leu3289Arg	Missense	Yes
			Patient 4	76557998	T	A	Tyr545Phe	Missense	Yes

MUC16	NM_024690.2	chr19	Patient 5	9057619	A	G	Ser9943Pro	Missense	Yes
			Patient 2	9077617	T	A	Met3277Leu	Missense	Yes

PTPN13	NM_080685.2	chr4	Patient 4	87694021	T	A	Tyr1758^∗∗∗^	Nonsese	Yes
			Patient 1	87656828	G	C	Glu745Gln	Missense	Yes

PCLO	NM_033026.5	chr7	Patient 4	82545516	T	A	Gln3929Leu	Missense	Yes
			Patient 1	82532009	T	A	Arg4496Trp	Missense	Yes

PDE1C	NM_001191058.1	chr7	Patient 4	31920370	C	A	Asp138Tyr	Missense	Yes
			Patient 2	32109908	C	T	Gly33Glu	Missense	Yes

^#^Coordinates refer to the human reference genome hg19 release (Genome Reference Consortium Human Build 37 (GRCh37), Feb. 2009); ^∗∗∗^stop codon.

## Abbreviations

eHCC: Early-stage hepatocellular carcinoma
HBV: Hepatitis B virus
SNVs: Single-nucleotide variants
indels: Small insertions and deletions
EMT: Epithelial-mesenchymal transition
ECM: Extracellular matrix.

## Supplementary Materials

Supplementary files Figure S1: The bioinformatic Flow chart for identification SNVs and Indels based on Illumina/Solexa sequencing. SNVs, single nucleotide variants; UTR, untranslated regions; dbSNP, single nucleotide polymorphism database; Indels, small insertions and deletions. Table S1: Summary of exome sequence analysis of 5 hepatocellular carcinomas. Table S2: The list of single-nucleotide variants discovered in five cases revealed by whole-exome sequencing. Table S3: The list of small insertions and deletions discovered in five cases revealed by whole-exome sequencing. Table S4: GO analysis of the biological processes of the affected SNVs. Table S5: Pathway analysis of the affected SNVs. Table S6: Primers used to carry out Sanger sequencing of the frequent mutations. Figure S2: The mutational signatures consisting of 96 different mutated trinucleotides identified in each case and all cases together. Signature 22 dominated in 4 out of 5 cases.
