# Supplementary material for: Whole-Exome Sequencing-Based Mutational Profiling of Hepatitis B Virus-Related Early-Stage Hepatocellular Carcinoma
Source: Gastroenterol Res Pract. 2017 Nov 29;2017:2029315. doi: 10.1155/2017/2029315 (PMC5733245; doi:10.1155/2017/2029315)
Supplement: Supplementary files — Figure S1: The bioinformatic Flow chart for identification SNVs and Indels based on Illumina/Solexa sequencing. SNVs, single nucleotide variants; UTR, untranslated regions; dbSNP, single nucleotide polymorphism database; Indels, small insertions and deletions. Table S1: Summary of exome sequence analysis of 5 hepatocellular carcinomas. Table S2: The list of single-nucleotide variants discovered in five cases revealed by whole-exome sequencing. Table S3: The list of small insertions and deletions discovered in five cases revealed by whole-exome sequencing. Table S4: GO analysis of the biological processes of the affected SNVs. Table S5: Pathway analysis of the affected SNVs. Table S6: Primers used to carry out Sanger sequencing of the frequent mutations. Figure S2: The mutational signatures consisting of 96 different mutated trinucleotides identified in each case and all cases together. Signature 22 dominated in 4 out of 5 cases. [file 2029315.f1.pdf]

Supplementary Figure 1.

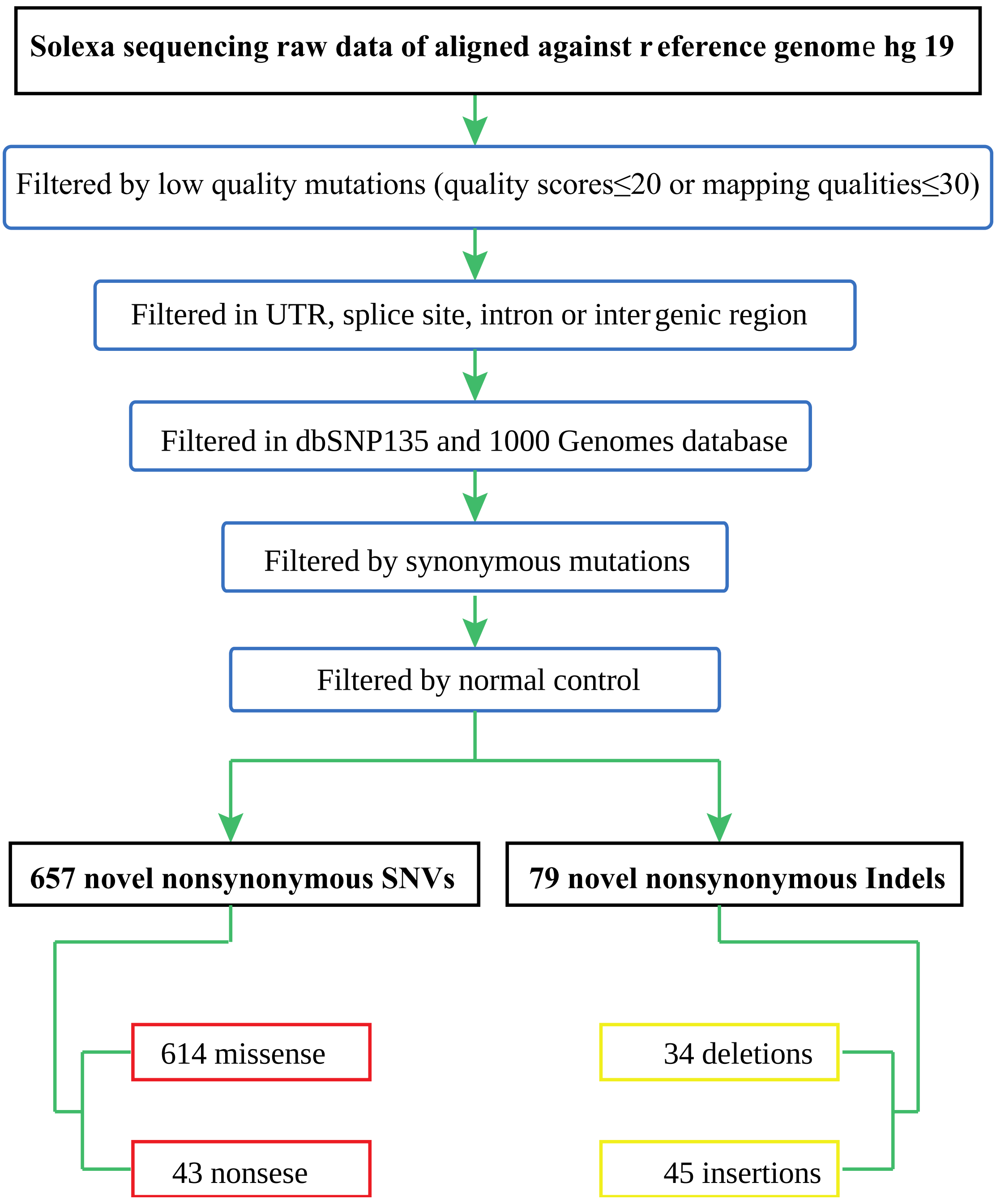

Supplementary Figure 1. The bioinformatic Flow chart for identification SNVs and Indels based on Illumina/Solexa sequencing. SNVs, single nucleotide variants; UTR, untranslated regions; dbSNP, single nucleotide polymorphism database; Indels, small insertions and deletions.

Supplementary Table 1. Summary of exome sequence analysis of 5 hepatocellular carcinomas

**Patient ID**	Patient 1		Patient 2		Patient 3		Patient 4		Patient 5		Averages
	Normal	Primary tumor	Normal	Primary tumor	Normal	Primary tumor	Normal	Primary tumor	Normal	Primary tumor	
**Mapped Reads**	67645664	72572267	72860802	84790105	83467110	80348956	59187866	74356456	65032298	66235825	73284927.25
**Uniquely Mapped Reads**	59732803	64036098	62037691	66346437	71163666	68312401	51978725	65534482	57108450	58472950	62619350.25
**Uniq/Mapped**	0.8830249	0.882377	0.851455	0.782479	0.8525953	0.8501965	0.878199	0.8813556	0.8781552	0.8827995	0.857154388
**Less than 2 mismatch(Good)**	59732803	64036098	62037650	66346437	71163629	68312335	51978725	65534482	57108450	58472950	62619332.25
**Good/Uniq**	1	1	0.9999993	1	0.9999995	0.999999	1	1	1	1	0.999999725
**Reads In Target**	47144439	49994664	48143139	51422166	55474802	52582294	40684668	48678700	44916061	45256206	48394754.5
**Reads In Target/Uniq**	0.7892554	0.7807263	0.7760305	0.775055	0.7795383	0.7697328	0.7827177	0.7427952	0.7865046	0.7739682	0.773292788
**Median Depth**	62	64	65	68	73	68	54	64	59	57	63.5
**Target area been covered**	0.9551259	0.9563305	0.9594348	0.957385	0.9578073	0.959752	0.9532092	0.9564446	0.953398	0.9551003	0.9565664
**Bases In Target**	3890943606	4118795544	3977836498	4250000000	4570763004	4353012495	3367085005	4015182136	3695382259	3725113573	3994296871
**Mean Depth in Target**	78.71049	83.21479	80.10686	85.78198	92.2038	87.6333	68.25023	81.11174	74.88993	75.3579	80.6669675

Abbreviation; Uniq, Uniquely Mapped Reads.

Supplementary Table 2. The list of single-nucleotide variants discovered in five cases revealed by whole-exome sequencing

**Sample**	**Gene**	**Gene ID**	**Chromosome**	**Position**	**Reference Base**	**Mutation Base**	**Convert Type**	**Mutation Type**
2T	ABCA12	NM_173076.2	chr2	215847037	T	C	Arg1485Gly	missense
5T	ABCA13	NM_152701.3	chr7	48352694	C	T	Leu3183Phe	missense
4T	ABCA3	NM_001089.2	chr16	2369715	T	G	Tyr247Ser	missense
5T	ABCD4	NM_005050.3	chr14	74757027	G	A	Arg432Trp	missense
2T	ABCG1	NM_207627.1	chr21	43710287	T	A	Leu453Gln	missense
1T	ACAN	NM_001135.3	chr15	89401182	C	T	Thr1789Ile	missense
2T	ACTL5T	NM_006686.3	chr9	111617055	T	C	Ile386Val	missense
5T	ADAM22	NM_021723.3	chr7	87810873	T	C	Ile821Thr	missense
5T	ADORA2T	NM_000676.2	chr17	15878550	G	A	Arg298His	missense
2T	AGAP10	XM_001714786.4	chr10	47192314	G	A	Thr602Ile	missense
3T	AGBL5	NM_021831.5	chr2	27276868	C	G	Cys164Trp	missense
5T	AJAP1	NM_018836.3	chr1	4772615	A	T	Met229Leu	missense
2T	AK2	NM_013411.4	chr1	33486978	T	C	Ile139Val	missense
5T	AKAP12	NM_005100.3	chr6	151671715	C	A	Ala730Asp	missense
5T	AKAP4	NM_003886.2	chrX	49958910	A	T	Ser152Thr	missense
5T	AKD1	NM_001145128.2	chr6	109993320	T	C	Met78Val	missense
3T	ALB	NM_000477.5	chr4	74274473	G	A	Asp145Asn	missense

Sample	Gene	Gene ID	Chromosome	Position	Reference Base	Mutation Base	Convert Type	Mutation Type
5T	ALG1T	NM_001013620.3	chr12	38714723	A	T	Tyr377Phe	missense
2T	ALPP	NM_001632.3	chr2	233245199	A	T	Met288Leu	missense
2T	ALPPL2	NM_031313.2	chr2	233273280	A	T	Met285Leu	missense
2T	AMDHD2	NM_001145815.1	chr16	2580437	T	G	Trp488Gly	missense
5T	ANAPC4	NM_013367.2	chr4	25419260	A	G	Ile700Val	missense
4T	ANKS1A	NM_015245.2	chr6	35053703	G	A	Arg1098Lys	missense
5T	ANO6	NM_001142679.1	chr12	45803211	A	T	Gln651Leu	missense
5T	APOB	NM_000384.2	chr2	21233653	T	A	Leu2029Phe	missense
3T	ARHGEF28	NM_001080479.2	chr5	73136356	A	T	Thr400Ser	missense
5T	ATP6AP2	NM_005765.2	chrX	40460032	A	T	Ser253Cys	missense
4T	ATP6V1B1	NM_001692.3	chr2	71187145	G	A	Met174Ile	missense
3T	B3GNT7	NM_145236.2	chr2	232263268	A	T	Asn280Tyr	missense
2T	BCAT2	NM_001190.3	chr19	49303278	C	T	Gly164Asp	missense
2T	BCL11A	NM_018014.3	chr2	60688947	G	C	Pro367Arg	missense
5T	BDP1	NM_018429.2	chr5	70858175	A	T	Gln2524Leu	missense
2T	BRCA2	NM_000059.3	chr13	32914130	A	T	Asn1880Tyr	missense
2T	BRWD1	NM_018963.4	chr21	40585423	T	A	Gln1281Leu	missense
5T	C17orf53	NM_024032.3	chr17	42225320	A	T	Gln50Leu	missense
2T	C1orf116	NM_023938.5	chr1	207196159	T	G	Asp317Ala	missense
4T	C1orf68	NM_001024679.2	chr1	152692640	T	A	Cys215Ser	missense
5T	C2orf74	NM_001143960.1	chr2	61390277	A	T	Leu28Phe	missense
4T	C3	NM_000064.2	chr19	6707877	C	G	Gly637Arg	missense
5T	C5	NM_001735.2	chr9	123776235	T	A	Ile725Phe	missense
1T	CACNA1E	NM_000721.3	chr1	181706764	G	A	Val1176Ile	missense
2T	CACNA1S	NM_000069.2	chr1	201039431	T	A	Ser777Cys	missense
5T	CACNA2D3	NM_018398.2	chr3	54919557	T	A	Leu667Gln	missense
4T	CACNB1	NM_000723.4	chr17	37341056	G	C	Pro237Arg	missense
2T	CAPSL	NM_144647.3	chr5	35909985	A	T	Tyr170Asn	missense
2T	CARNS1	NM_001166222.1	chr11	67191118	C	A	His633Gln	missense
2T	CASP7	NM_033340.3	chr10	115457335	G	A	Arg28Gln	missense
2T	CASP8AP2	NM_012115.3	chr6	90576988	A	T	Ser1327Cys	missense
5T	CASQ2	NM_001232.3	chr1	116311062	A	G	Val34Ala	missense
2T	CCDC108	NM_194302.2	chr2	219873863	T	A	Gln1527Leu	missense
2T	CCDC114	NM_144577.3	chr19	48815274	T	C	Ile117Val	missense
5T	CCDC141	NM_173648.3	chr2	179839845	A	T	His155Gln	missense
5T	CCDC175	NM_001164399.1	chr14	60004858	T	A	Gln502His	missense
3T	CCDC73	NM_001008391.2	chr11	32635262	A	T	Ser868Thr	missense
2T	CCDC79	NM_001136505.1	chr16	66830682	T	A	Lys7Met	missense
5T	CCR4	NM_005508.4	chr3	32995557	T	A	Leu215Ile	missense
5T	CD164L2	NM_207397.2	chr1	27706604	A	T	Leu152Gln	missense
5T	CD300LD	NM_001115152.1	chr17	72584946	T	A	Asn28Ile	missense
2T	CDHR5	NM_031264.3	chr11	618051	A	C	Val480Gly	missense

Sample	Gene	Gene ID	Chromosome	Position	Reference Base	Mutation Base	Convert Type	Mutation Type
2T	CDK13	NM_031267.3	chr7	40127790	G	T	Gly1032Val	missense
4T	CDK17	NM_001170464.2	chr12	96707193	T	C	Glu108Gly	missense
2T	CENPK	NM_001267038.1	chr5	64847403	T	C	Lys77Glu	missense
5T	CEP85L	NM_001178035.1	chr6	118844968	T	A	Asp382Val	missense
4T	CFH	NM_000186.3	chr1	196697616	A	G	Ile793Val	missense
2T	CHD5	NM_015557.2	chr1	6202559	T	A	Gln717Leu	missense
2T	CHIT1	NM_001256125.1	chr1	203188878	A	T	Ser258Thr	missense
3T	CHRDL2	NM_015424.3	chr11	74408263	A	G	Trp419Arg	missense
5T	CILP	NM_003613.3	chr15	65491375	A	T	Cys417Ser	missense
4T	CKAP4	NM_006825.3	chr12	106632912	T	C	Ile567Val	missense
5T	CLCN3	NM_173872.3	chr4	170625293	A	T	Thr570Ser	missense
5T	CLEC4F	NM_001258027.1	chr2	71043579	T	A	Asn312Tyr	missense
5T	CLIP2	NM_032421.2	chr7	73790701	A	T	Lys622Met	missense
5T	CLSTN2	NM_022131.2	chr3	140265416	A	T	Thr523Ser	missense
5T	CMPK2	NM_001256478.1	chr2	7001453	G	T	Pro285His	missense
2T	CNGB3	NM_019098.4	chr8	87616357	A	G	Val582Ala	missense
5T	CNR2	NM_001841.2	chr1	24201971	A	T	Leu46Gln	missense
4T	CNTF	NM_000614.3	chr11	58391631	A	T	Tyr80Phe	missense
5T	COA6	NM_001012985.2	chr1	234509574	C	A	Ser37Tyr	missense
2T	COL11A2	NM_080681.2	chr6	33132099	T	C	Asp1586Gly	missense
5T	COL4A6	NM_001847.2	chrX	107457397	T	A	Gln130Leu	missense
5T	COL5A1	NM_000093.3	chr9	137713952	G	T	Gly1522Cys	missense
5T	COL6A3	NM_004369.3	chr2	238270466	A	C	Ile2024Met	missense
5T	COL6A3	NM_004369.3	chr2	238277313	T	A	Glu1598Val	missense
5T	COL6A5	NM_153264.5	chr3	130159321	A	G	Thr2047Ala	missense
3T	COL7A1	NM_000094.3	chr3	48621194	C	A	Gly1433Val	missense
1T	COMMD7	NM_001099339.1	chr20	31294506	C	A	Gly99Cys	missense
5T	COPS5	NM_006837.2	chr8	67963508	T	C	Asn243Ser	missense
5T	CR1L	NM_175710.1	chr1	207868029	A	T	Lys265Asn	missense
4T	CRADD	NM_003805.3	chr12	94243823	A	T	Arg126Trp	missense
5T	CRHR1	NM_001256299.1	chr17	43911202	T	A	Leu176Gln	missense
5T	CRHR2	NM_001202475.1	chr7	30693080	A	T	Val438Glu	missense
2T	CRTC3	NM_001042574.2	chr15	91147646	A	T	His148Leu	missense
2T	CSMD1	NM_033225.5	chr8	3265724	C	A	Ala590Ser	missense
2T	CSPG5	NM_001206945.1	chr3	47604141	T	A	Lys412Asn	missense
5T	CTBP2	NM_001083914.1	chr10	126691999	C	T	Gly41Ser	missense
5T	CTSL1	NM_145918.2	chr9	90343504	A	T	Gln134Leu	missense
5T	CTU2	NM_001012762.1	chr16	88776369	T	G	Val56Gly	missense
4T	CUBN	NM_001081.3	chr10	16996493	C	T	Glu1584Lys	missense
5T	CYFIP1	NM_014608.2	chr15	22933656	A	T	Gln222Leu	missense
5T	CYLD	NM_015247.2	chr16	50825480	A	C	Gln707Pro	missense
4T	CYP2F1	NM_000774.3	chr19	41633809	G	A	Arg433His	missense

Sample	Gene	Gene ID	Chromosome	Position	Reference Base	Mutation Base	Convert Type	Mutation Type
4T	CYP4F11	NM_021187.3	chr19	16033183	T	C	Met326Val	missense
2T	DAZ1	NM_004081.5	chrY	25308542	G	T	Pro553Gln	missense
2T	DAZ2	NM_001005785.2	chrY	25380604	C	A	Pro223Gln	missense
2T	DAZ3	NM_020364.3	chrY	26944616	G	T	Pro223Gln	missense
2T	DAZ4	NM_020420.3	chrY	27005838	C	A	Pro223Gln	missense
5T	DBF4	NM_006716.3	chr7	87537275	A	T	Asn608Tyr	missense
4T	DCAF12L2	NM_001013628.2	chrX	125298898	C	T	Arg337His	missense
4T	DCC	NM_005215.3	chr18	50705426	T	A	Tyr505Asn	missense
3T	DCDC2T	NM_001099434.1	chr1	32678374	T	A	Ser232Thr	missense
5T	DCHS2	NM_001142552.1	chr4	155298524	A	T	Tyr709Asn	missense
4T	DCLRE1CP1	XM_002342972.1	chr10	15057454	A	T	Leu102Gln	missense
5T	DDC	NM_001082971.1	chr7	50571745	G	C	Leu243Val	missense
2T	DDHD1	NM_001160147.1	chr14	53558512	T	C	Asn434Ser	missense
2T	DEK	NM_003472.3	chr6	18258248	A	T	Leu98Gln	missense
4T	DEPDC1	NM_017779.4	chr1	68960320	T	A	His36Leu	missense
5T	DIDO1	NM_033081.2	chr20	61522506	T	A	Glu1116Val	missense
5T	DIRAS1	NM_145173.3	chr19	2717398	T	A	Gln136Leu	missense
4T	DISC1	NM_001164549.1	chr1	231906660	A	T	Gln493Leu	missense
4T	DMD	NM_000109.3	chrX	32834714	G	T	Thr126Asn	missense
1T	DMRT1	NM_021951.2	chr9	968077	T	C	Cys354Arg	missense
4T	DNAH11	NM_003777.3	chr7	21847619	T	C	Ser3436Pro	missense
5T	DNAH17	NM_173628.3	chr17	76454758	A	C	Leu3289Arg	missense
4T	DNAH17	NM_173628.3	chr17	76557998	T	A	Tyr545Phe	missense
4T	DNAH5	NM_001369.2	chr5	13708416	G	A	Pro4385Leu	missense
5T	DNAH9	NM_001372.3	chr17	11592983	T	A	Phe1282Ile	missense
3T	DNAJC16	NM_015291.2	chr1	15890443	G	A	Arg453His	missense
1T	DNAJC6	NM_001256865.1	chr1	65867465	A	G	Lys640Arg	missense
5T	DNM1	NM_001005336.1	chr9	130984835	A	T	Asn363Ile	missense
4T	DNMBP	NM_015221.2	chr10	101715862	C	A	Ala457Ser	missense
2T	DOCK3	NM_004947.4	chr3	51264786	A	T	Ser484Cys	missense
5T	DOCK8	NM_203447.3	chr9	420425	A	T	Asn1289Tyr	missense
2T	DOCK9	NM_015296.2	chr13	99550460	T	C	Tyr521Cys	missense
2T	DPP10	NM_020868.3	chr2	116572444	T	A	Asp592Glu	missense
5T	DPPA4	NM_018189.3	chr3	109049382	T	A	Gln223Leu	missense
2T	DROSHA	NM_013235.4	chr5	31511262	T	A	Thr438Ser	missense
2T	DSC1	NM_024421.2	chr18	28721992	A	T	Asn410Lys	missense
5T	DST	NM_015548.4	chr6	56335990	C	A	Arg4789Leu	missense
5T	DUX4	NM_033178.2	chr4	191006807	A	T	Glu475Val	missense
5T	DZIP3	NM_014648.3	chr3	108366878	G	T	Glu627Asp	missense
5T	EEA1	NM_003566.3	chr12	93196191	T	C	Ile887Val	missense
1T	EEF1A1	NM_001402.5	chr6	74229205	A	G	Leu60Ser	missense
3T	EEF1A2	NM_001958.3	chr20	62121960	C	T	Glu301Lys	missense

Sample	Gene	Gene ID	Chromosome	Position	Reference Base	Mutation Base	Convert Type	Mutation Type
5T	EMB	NM_198449.2	chr5	49701574	A	T	Ser195Arg	missense
4T	EMC3	NM_018447.2	chr3	10005842	C	T	Ala233Thr	missense
2T	ENAM	NM_031889.2	chr4	71508252	A	C	Gln370Pro	missense
4T	EPHA1	NM_005232.4	chr7	143092248	A	C	Phe704Cys	missense
5T	EPHA6	NM_001080448.2	chr3	97356838	T	A	Leu899Gln	missense
5T	ERMN	NM_001009959.1	chr2	158182036	A	T	Leu53Gln	missense
4T	EXT2	NM_000401.3	chr11	44129243	A	T	Glu27Val	missense
5T	EYS	NM_001142800.1	chr6	65327357	A	T	Met1213Lys	missense
5T	EYS	NM_001142800.1	chr6	66044877	G	A	Pro588Ser	missense
5T	FAM124A	NM_145019.3	chr13	51854859	G	A	Gly406Ser	missense
4T	FAM134C	NM_178126.3	chr17	40739858	T	G	Asn124Thr	missense
5T	FAM13A	NM_014883.3	chr4	89711773	T	A	His352Leu	missense
5T	FAM198B	NM_001031700.2	chr4	159048658	T	A	Gln495His	missense
5T	FAM58BP	NM_001105517.1	chr1	200183089	T	A	Leu133Gln	missense
5T	FAT2	NM_001447.2	chr5	150922378	A	T	His2770Gln	missense
3T	FBF1	NM_001080542.1	chr17	73929103	A	T	Met33Lys	missense
5T	FLT1	NM_002019.4	chr13	29012453	T	A	Ser140Cys	missense
5T	FLT3	NM_004119.2	chr13	28623523	A	C	Val345Gly	missense
2T	FMO1	NM_002021.1	chr1	171252338	A	T	Lys413Asn	missense
5T	FOLR4	NM_001199206.1	chr11	94039854	C	T	Pro105Leu	missense
5T	FOLR4	NM_001199206.1	chr11	94039796	T	A	Cys86Ser	missense
2T	FOXN1	NM_003593.2	chr17	26862078	A	C	Thr497Pro	missense
5T	FOXP2	NM_014491.3	chr7	114174760	A	T	Gln86Leu	missense
5T	FPR1	NM_001193306.1	chr19	52249387	A	T	Ser287Arg	missense
5T	FRAS1	NM_025074.6	chr4	79394648	A	T	Met2527Leu	missense
2T	FRMD7	NM_194277.2	chrX	131228165	T	G	Tyr96Ser	missense
4T	FRRS1	NM_001013660.2	chr1	100181207	T	A	Thr420Ser	missense
4T	FSTL1	NM_007085.4	chr3	120128461	T	A	Gln127Leu	missense
4T	FZD10	NM_007197.3	chr12	130648716	C	T	Thr410Met	missense
2T	GABPB2	NM_144618.2	chr1	151089873	A	T	Thr310Ser	missense
4T	GALNT13	NM_052917.2	chr2	155157974	A	T	His343Leu	missense
2T	GARNL3	NM_032293.4	chr9	130155519	A	G	Ile1010Val	missense
5T	GCAT	NM_001171690.1	chr22	38208984	G	T	Gly166Cys	missense
5T	GCNT4	NM_016591.2	chr5	74324571	T	A	Gln431Leu	missense
2T	GDF6	NM_001001557.2	chr8	97172832	G	A	Ser30Phe	missense
2T	GGT2	XM_003846303.1	chr22	21567365	C	T	Asp342Asn	missense
2T	GIT2	NM_057169.3	chr12	110383065	C	A	Arg577Ser	missense
2T	GJB2	NM_004004.5	chr13	20763525	C	T	Asp66Asn	missense
5T	GLI2	NM_005270.4	chr2	121748249	T	A	***1587Lys	missense
5T	GLI3	NM_000168.5	chr7	42004682	T	A	Asp1330Val	missense
5T	GON4L	NM_001037533.1	chr1	155786014	T	A	Asn352Tyr	missense
5T	GPAM	NM_020918.5	chr10	113933521	C	T	Val166Met	missense

Sample	Gene	Gene ID	Chromosome	Position	Reference Base	Mutation Base	Convert Type	Mutation Type
5T	GPR18	NM_001098200.1	chr13	99907901	G	A	Pro76Ser	missense
3T	GPR37	NM_005302.2	chr7	124386752	T	A	Ser557Cys	missense
5T	GPR82	NM_080817.4	chrX	41587169	T	A	Leu297His	missense
5T	GPR98	NM_032119.3	chr5	90040905	G	C	Cys3531Ser	missense
5T	GPR98	NM_032119.3	chr5	90040895	G	T	Ala3528Ser	missense
4T	GPRC6A	NM_148963.2	chr6	117114150	T	A	Ser646Cys	missense
1T	GRHL1	NM_198182.2	chr2	10095090	C	T	Arg23Trp	missense
5T	GRM7	NM_181874.2	chr3	7620328	A	T	Ile579Phe	missense
4T	GTF2A1	NM_201595.2	chr14	81670430	T	A	Met12Leu	missense
5T	GTF2H4	NM_001517.4	chr6	30876821	G	T	Ser3Ile	missense
5T	GTF3C1	NM_001520.3	chr16	27509997	T	C	Ile707Val	missense
4T	GUSB	NM_000181.3	chr7	65444505	T	C	Gln202Arg	missense
2T	HAPLN3	NM_178232.2	chr15	89424716	C	T	Arg122Gln	missense
4T	HCAR3	NM_006018.2	chr12	123200723	A	C	Trp188Gly	missense
5T	HECTD1	NM_015382.2	chr14	31582610	T	G	Leu1979Phe	missense
5T	HECTD2	NM_182765.3	chr10	93220217	G	A	Arg101Lys	missense
5T	HEMGN	NM_018437.3	chr9	100700380	A	T	His13Gln	missense
5T	HEPH	NM_138737.3	chrX	65420426	T	A	Cys691Ser	missense
5T	HHLA2	NM_007072.2	chr3	108081293	G	A	Ala370Thr	missense
5T	HIBADH	NM_152740.3	chr7	27565891	T	A	Tyr318Phe	missense
5T	HK3	NM_002115.2	chr5	176318406	G	A	Ser81Phe	missense
2T	HMGCS2	NM_001166107.1	chr1	120311383	G	A	Leu29Phe	missense
4T	HOXC10	NM_017409.3	chr12	54379192	C	A	Ala50Glu	missense
4T	HOXD8	NM_001199746.1	chr2	176995464	T	A	Cys124Ser	missense
5T	HRC	NM_002152.2	chr19	49657139	A	T	His452Gln	missense
5T	HS3ST2	NM_006043.1	chr16	22926554	A	T	Ser259Cys	missense
1T	HS3ST3A1	NM_006042.1	chr17	13504386	T	C	Ile21Val	missense
2T	HSDL2	NM_001195822.1	chr9	115216360	A	T	Glu238Asp	missense
5T	HSFX1	NM_016153.2	chrX	148856862	C	T	Arg175Cys	missense
5T	HSFX2	NM_001164415.1	chrX	148675838	G	A	Arg175Cys	missense
5T	HSFY1	NM_033108.2	chrY	20709873	C	A	Ala241Asp	missense
5T	HSFY2	NM_153716.1	chrY	20934305	G	T	Ala241Asp	missense
2T	HSPA2	NM_021979.3	chr14	65007614	A	T	Tyr16Phe	missense
2T	HTR6	NM_000871.1	chr1	20005493	A	G	Ile319Val	missense
5T	HVCN1	NM_001256413.1	chr12	111089121	A	T	Phe162Ile	missense
5T	IARS	NM_002161.4	chr9	94985694	T	A	Ile1163Phe	missense
4T	IBTK	NM_015525.2	chr6	82920555	T	A	Thr829Ser	missense
2T	IDH2	NM_002168.2	chr15	90627553	G	T	Thr435Lys	missense
4T	Ighv1-58	IGHV1-58	chr14	107078491	T	C	Asn78Ser	missense
1T	IGSF3	NM_001542.2	chr1	117146585	G	A	Arg449Cys	missense
4T	IL1B	NM_000576.2	chr2	113588132	A	T	Tyr206Asn	missense
5T	IL4R	NM_001257997.1	chr16	27374766	A	T	Gln538Leu	missense

Sample	Gene	Gene ID	Chromosome	Position	Reference Base	Mutation Base	Convert Type	Mutation Type
2T	INADL	NM_176877.2	chr1	62228831	A	G	Ile57Val	missense
2T	INMT	NM_001199219.1	chr7	30793553	A	T	Ser120Cys	missense
4T	IPO11	NM_016338.4	chr5	61779876	T	C	Met354Thr	missense
2T	IREB2	NM_004136.2	chr15	78782801	A	T	Asp705Val	missense
2T	ISLR2	NM_001130136.1	chr15	74425160	A	T	Glu22Val	missense
4T	ITGA8	NM_003638.1	chr10	15559209	T	A	Glu1047Val	missense
2T	ITIH3	NM_002217.3	chr3	52835020	A	T	Asn414Ile	missense
4T	ITK	NM_005546.3	chr5	156635906	C	A	Arg49Ser	missense
1T	ITPR3	NM_002224.3	chr6	33636859	G	T	Glu705Asp	missense
1T	ITPR3	NM_002224.3	chr6	33636860	A	G	Lys706Glu	missense
4T	JAG2	NM_002226.4	chr14	105609472	C	A	Val1093Leu	missense
2T	KAL1	NM_000216.2	chrX	8522042	G	T	Phe435Leu	missense
4T	KAT2A	NM_021078.2	chr17	40269496	A	C	Val516Gly	missense
5T	KCNC2	NM_139136.3	chr12	75444332	C	T	Ala485Thr	missense
5T	KCNG4	NM_172347.2	chr16	84255889	A	T	His498Gln	missense
2T	KCNMB1	NM_004137.2	chr5	169805957	G	T	Ser109Arg	missense
1T	KDM3T	NM_016604.3	chr5	137762930	G	T	Gly1519Cys	missense
5T	KDR	NM_002253.2	chr4	55948180	A	T	Leu1264His	missense
5T	KIAA0408	NM_014702.4	chr6	127771397	T	A	Glu79Val	missense
5T	KIAA0825	NM_001145678.1	chr5	93856414	C	A	Arg170Leu	missense
5T	KIAA0922	NM_015196.3	chr4	154507490	A	T	Ile480Phe	missense
4T	KIAA1715	NM_030650.1	chr2	176844586	T	A	Ile90Leu	missense
1T	KIF16B	NM_001199865.1	chr20	16360128	T	C	Glu840Gly	missense
5T	KIF18A	NM_031217.3	chr11	28119181	T	C	Tyr105Cys	missense
3T	KIF2T	NM_032559.4	chr17	51901982	A	T	Thr530Ser	missense
5T	KIF3C	NM_002254.6	chr2	26203838	A	T	Tyr317Asn	missense
4T	KIR2DS4	NM_012314.3	chr19	55351072	G	T	Gly187Val	missense
5T	KIRREL2	NM_199179.2	chr19	36349762	A	T	His123Leu	missense
5T	KLHL33	NM_001109997.2	chr14	20897961	T	A	Thr292Ser	missense
5T	KLHL40	NM_152393.2	chr3	42729779	T	A	Met433Lys	missense
3T	KRT12	NM_000223.3	chr17	39023072	C	T	Gly123Arg	missense
5T	KRT2	NM_000423.2	chr12	53042069	A	G	Met337Thr	missense
4T	KRT33A	NM_004138.3	chr17	39506947	T	G	Ser25Arg	missense
1T	KRTAP11-1	NM_175858.2	chr21	32253626	C	A	Arg73Leu	missense
1T	KRTCAP3	NM_001168364.1	chr2	27666029	T	C	Val121Ala	missense
5T	L3MBTL1	NM_032107.4	chr20	42164528	A	C	Tyr604Ser	missense
3T	LAMA1	NM_005559.3	chr18	7037735	C	T	Gly527Arg	missense
2T	LAMA2	NM_000426.3	chr6	129674494	A	T	Glu1570Val	missense
2T	LAMA3	NM_198129.1	chr18	21530076	G	A	Gly3199Arg	missense
4T	LAMB1	NM_002291.2	chr7	107626482	T	A	Ser221Cys	missense
2T	LAMC1	NM_002293.3	chr1	183091228	G	C	Gly748Ala	missense
5T	LAMP3	NM_014398.3	chr3	182871697	T	C	Lys178Glu	missense

Sample	Gene	Gene ID	Chromosome	Position	Reference Base	Mutation Base	Convert Type	Mutation Type
5T	LCE3E	NM_178435.2	chr1	152538480	T	A	Asn69Tyr	missense
2T	LCP2	NM_005565.3	chr5	169693876	G	T	Asp236Glu	missense
5T	LDB1	NM_001113407.1	chr10	103870305	C	T	Gly169Asp	missense
3T	LILRA6	NM_024318.2	chr19	54744797	A	T	Ser289Thr	missense
4T	LIMS3	NM_033514.4	chr2	110660898	C	A	Thr84Lys	missense
4T	LMAN1L	NM_021819.2	chr15	75112440	G	T	Glu258Asp	missense
4T	LMLN	NM_033029.3	chr3	197707210	A	T	Tyr188Phe	missense
3T	LOC100287294	XM_002342726.2	chr7	57009074	G	C	Ser77Cys	missense
3T	LOC402269	XM_377941.7	chr7	63095970	C	G	Ser77Cys	missense
2T	LOC642643	XM_003403485.1	chr22	18721555	G	T	Gly6Cys	missense
2T	LOC728888	XM_002343430.4	chr16	29401427	T	G	Lys127Thr	missense
2T	LPHN3	NM_015236.4	chr4	62897177	C	A	Ala1079Glu	missense
4T	LRFN5	NM_152447.3	chr14	42356294	T	A	Tyr156Asn	missense
5T	LRP2	NM_004525.2	chr2	170103464	T	A	Asn981Tyr	missense
2T	LRRC33	NM_198565.1	chr3	196388375	T	A	Cys621Ser	missense
5T	LRRC35T	NM_052888.2	chr17	30349349	A	T	Glu395Val	missense
2T	LRRC4B	NM_001080457.1	chr19	51020935	T	A	Ser679Cys	missense
5T	LRRC52	NM_001005214.3	chr1	165514089	T	A	Trp186Arg	missense
5T	LRRK2	NM_198578.3	chr12	40745356	A	T	Asn2133Tyr	missense
5T	LRRTM1	NM_178839.4	chr2	80529549	T	A	Thr466Ser	missense
2T	MACC1	NM_182762.3	chr7	20199394	G	T	Thr197Lys	missense
5T	MAGEB5	XM_003846439.1	chrX	26236211	A	T	Arg313Trp	missense
5T	MAGI3	NM_001142782.1	chr1	114225699	A	C	Lys1170Thr	missense
2T	MAN2C1	NM_006715.3	chr15	75656903	C	T	Ala176Thr	missense
2T	MAP2	NM_002374.3	chr2	210560692	G	C	Arg1266Ser	missense
5T	MAP3K19	NM_025052.3	chr2	135745799	A	T	Leu215Met	missense
5T	MAP7D3	NM_001173516.1	chrX	135326810	T	G	Gln115Pro	missense
5T	MAST4	NM_001164664.1	chr5	66462193	A	T	Ser2396Cys	missense
3T	MAX	NM_145112.1	chr14	65560436	T	C	Gln45Arg	missense
4T	MCM6	NM_005915.5	chr2	136616959	C	A	Ser425Ile	missense
2T	MDFI	NM_005586.3	chr6	41613983	G	A	Gly66Ser	missense
5T	MDN1	NM_014611.1	chr6	90395602	T	A	Gln3888Leu	missense
5T	MED12	NM_005120.2	chrX	70342071	A	T	Ser375Cys	missense
5T	MED17	NM_004268.4	chr11	93535071	A	G	Ile467Val	missense
5T	MEF2C	NM_001131005.2	chr5	88047839	C	T	Glu140Lys	missense
1T	MET	NM_001127500.1	chr7	116423433	T	G	Ser1254Arg	missense
4T	METTL11B	NM_001136107.1	chr1	170129724	C	A	Leu74Ile	missense
1T	METTL17	NM_022734.2	chr14	21464731	C	T	Arg376Trp	missense
5T	MFRP	NM_031433.2	chr11	119213336	T	A	Asn453Tyr	missense
4T	MGAM	NM_004668.2	chr7	141754622	C	G	Ser1076Arg	missense
2T	MINOS1	NM_001032363.3	chr1	19923540	A	T	Met1Leu	missense
3T	MKNK2	NM_017572.3	chr19	2037827	A	C	Trp386Gly	missense

Sample	Gene	Gene ID	Chromosome	Position	Reference Base	Mutation Base	Convert Type	Mutation Type
5T	MLL	NM_001197104.1	chr11	118355607	A	T	Met1417Leu	missense
2T	MLLT4	NM_001207008.1	chr6	168352601	T	C	Trp1499Arg	missense
2T	MMP10	NM_002425.2	chr11	102650307	C	A	Gly92Val	missense
2T	MMP27	NM_022122.2	chr11	102565741	T	A	Gln330His	missense
5T	MMS22L	NM_198468.2	chr6	97729122	T	A	His94Leu	missense
5T	MORC2	NM_014941.1	chr22	31335674	T	A	Gln274Leu	missense
2T	MPG	NM_001015054.2	chr16	135664	T	G	Val245Gly	missense
4T	MRGPRX1	NM_147199.3	chr11	18955433	T	C	Glu300Gly	missense
5T	MRPL14	NM_032111.2	chr6	44081884	G	A	Ala45Val	missense
5T	MSH3	NM_002439.4	chr5	80063903	A	T	Glu683Val	missense
3T	MST1	NM_020998.3	chr3	49724183	C	G	Glu261Gln	missense
2T	MTNR1B	NM_005959.3	chr11	92715191	A	T	Asn268Tyr	missense
2T	MUC16	NM_024690.2	chr19	9077617	T	A	Met3277Leu	missense
5T	MUC16	NM_024690.2	chr19	9057619	A	G	Ser9943Pro	missense
5T	MUC21	NM_001010909.2	chr6	30955136	C	G	Ser395Cys	missense
5T	MUC4	NM_138297.4	chr3	195488420	A	T	Met563Lys	missense
5T	MUC5AC	XM_003403450.2	chr11	1217632	A	T	Asp1308Val	missense
5T	MYBPH	NM_004997.2	chr1	203144741	T	A	Gln48Leu	missense
5T	MYCBP2	NM_015057.4	chr13	77631149	A	C	Phe4470Cys	missense
5T	MYH1	NM_005963.3	chr17	10400466	G	A	Arg1526Cys	missense
5T	MYH1	NM_005963.3	chr17	10397732	T	A	Gln1869Leu	missense
4T	MYH4	NM_017533.2	chr17	10359172	T	A	His672Leu	missense
2T	MYL10	NM_138403.4	chr7	101266339	A	T	Met96Lys	missense
5T	MYO10	NM_012334.2	chr5	16762217	G	C	Asn531Lys	missense
5T	MYO3A	NM_017433.4	chr10	26417420	G	A	Glu739Lys	missense
2T	MYO3T	NM_138995.4	chr2	171056760	A	T	His96Leu	missense
1T	MYO5A	NM_000259.3	chr15	52700306	T	C	Tyr263Cys	missense
5T	MYO5C	NM_018728.3	chr15	52527944	T	C	Gln962Arg	missense
5T	NAALAD2	NM_005467.3	chr11	89880640	A	C	Thr113Pro	missense
5T	NEB	NM_001164507.1	chr2	152527725	A	T	Tyr1440Asn	missense
5T	NEB	NM_001164507.1	chr2	152500450	T	A	Lys2613Met	missense
4T	NEDD4L	NM_001243960.1	chr18	56050528	T	G	Asn737Lys	missense
5T	NLGN1	NM_014932.2	chr3	173996939	T	C	Ile383Thr	missense
5T	NLRP10	NM_176821.3	chr11	7982162	G	T	Gln333Lys	missense
5T	NLRP2	NM_001174081.1	chr19	55512228	T	A	Trp1051Arg	missense
4T	NOS3	NM_000603.4	chr7	150707224	T	G	Val845Gly	missense
3T	NRCAM	NM_005010.4	chr7	107824881	T	A	Gln722Leu	missense
2T	NRP1	NM_001244972.1	chr10	33515124	T	G	Lys359Gln	missense
2T	NTNG1	NM_001113226.1	chr1	107866950	A	T	Glu98Val	missense
3T	NUP210	NM_024923.2	chr3	13401858	C	T	Ser689Asn	missense
5T	NUP88	NM_002532.4	chr17	5292263	G	A	Ala501Val	missense
1T	OASL	NM_001261825.1	chr12	121471398	T	C	Gln116Arg	missense

Sample	Gene	Gene ID	Chromosome	Position	Reference Base	Mutation Base	Convert Type	Mutation Type
5T	OBP2T	NM_014581.2	chr9	136082651	T	A	Gln117Leu	missense
2T	OBSCN	NM_001098623.1	chr1	228430947	C	G	Ala998Gly	missense
5T	OLFM4	NM_006418.4	chr13	53603008	T	C	Phe13Leu	missense
4T	OPN4	NM_033282.3	chr10	88417867	G	T	Gln123His	missense
2T	OR10A4	NM_207186.2	chr11	6897997	T	C	Leu40Ser	missense
5T	OR10H4	NM_001004465.1	chr19	16060382	G	T	Ala189Ser	missense
5T	OR11G2	NM_001005503.1	chr14	20665952	T	A	Leu153Gln	missense
5T	OR13G1	NM_001005487.1	chr1	247835680	T	C	Ile222Val	missense
4T	OR14A16	NM_001001966.1	chr1	247978737	C	A	Val99Phe	missense
2T	OR2AG1	NM_001004489.2	chr11	6806791	A	T	Arg175Trp	missense
2T	OR4C12	NM_001005270.2	chr11	50003429	A	T	Phe203Leu	missense
5T	OR51G2	NM_001005238.1	chr11	4936186	C	A	Arg236Ser	missense
5T	OR51S1	NM_001004758.1	chr11	4870126	A	T	Cys105Ser	missense
2T	OR52L1	NM_001005173.2	chr11	6007629	G	T	Pro178Thr	missense
5T	OR52N1	NM_001001913.1	chr11	5809163	T	C	Tyr295Cys	missense
2T	OR56B4	NM_001005181.1	chr11	6129860	C	G	His284Gln	missense
3T	OR4T2	NM_001005566.2	chr11	58189884	T	C	Asn284Ser	missense
2T	OR5D14	NM_001004735.1	chr11	55563546	G	T	Gly172Val	missense
5T	OR6B3	NM_173351.1	chr2	240985266	G	C	Ser75Cys	missense
5T	OR6C68	NM_001005519.2	chr12	55887023	T	C	Tyr288His	missense
2T	OR8J1	NM_001005205.2	chr11	56128113	C	A	Leu131Met	missense
1T	OR8K3	NM_001005202.1	chr11	56086072	G	A	Cys97Tyr	missense
3T	OTOGL	NM_173591.3	chr12	80651707	A	T	Gln596Leu	missense
2T	OTOP3	NM_178233.1	chr17	72943380	T	G	Leu477Arg	missense
5T	OTUD4	NM_001102653.1	chr4	146062554	T	C	Gln622Arg	missense
4T	P2RX1	NM_002558.2	chr17	3808612	T	A	Ser63Cys	missense
2T	P2RY1	NM_002563.3	chr3	152554274	G	T	Gly235Cys	missense
3T	P4HTM	NM_177938.2	chr3	49042521	C	A	Pro372Gln	missense
4T	PAPPA2	NM_020318.2	chr1	176661372	A	T	Ile848Phe	missense
1T	PARD3T	NM_057177.6	chr2	205986575	C	A	Pro356Gln	missense
2T	PARK7	NM_007262.4	chr1	8045007	A	T	Ser155Cys	missense
4T	PARVA	NM_018222.4	chr11	12499457	G	A	Glu161Lys	missense
5T	PAX6	NM_001258462.1	chr11	31811521	A	T	Ser424Arg	missense
5T	PBLD	NM_022129.3	chr10	70048315	C	A	Gly206Trp	missense
2T	PCDH15	NM_001142772.1	chr10	55566637	G	A	Ala1579Val	missense
2T	PCDHA2	NM_031495.1	chr5	140176141	T	G	Leu531Arg	missense
2T	PCDHA2	NM_031495.1	chr5	140176684	T	A	Val712Glu	missense
5T	PCDHB1	NM_013340.2	chr5	140431804	A	T	Gln250Leu	missense
5T	PCDHGA1	NM_018912.2	chr5	140711060	A	T	Asp270Val	missense
1T	PCDHGA8	NM_014004.2	chr5	140774533	G	T	Arg718Leu	missense
2T	PCDHGB5	NM_032099.1	chr5	140778787	A	T	Ile365Phe	missense
4T	PCLO	NM_033026.5	chr7	82545516	T	A	Gln3929Leu	missense

Sample	Gene	Gene ID	Chromosome	Position	Reference Base	Mutation Base	Convert Type	Mutation Type
3T	PCLO	NM_033026.5	chr7	82532009	T	A	Arg4496Trp	missense
5T	PCNXL3	NM_032223.2	chr11	65386239	G	A	Arg469Lys	missense
5T	PCSK5	NM_001190482.1	chr9	78854054	A	T	Thr1016Ser	missense
2T	PDE1C	NM_001191057.1	chr7	32109908	C	T	Gly33Glu	missense
4T	PDE1C	NM_001191058.1	chr7	31920370	C	A	Asp138Tyr	missense
2T	PDE3T	NM_000922.3	chr11	14839756	A	C	Asn517Thr	missense
5T	PDK4	NM_002612.3	chr7	95221870	T	C	His190Arg	missense
5T	PDZD11	NM_016484.4	chrX	69509172	T	A	Tyr7Phe	missense
2T	PEG10	NM_015068.3	chr7	94294422	G	C	***518Tyr	missense
5T	PET112	NM_004564.2	chr4	152592445	C	T	Val519Met	missense
4T	PHC3	NM_024947.3	chr3	169815109	A	C	Leu966Arg	missense
4T	PHC3	NM_024947.3	chr3	169835203	A	C	Asp668Glu	missense
2T	PHF1	NM_024165.2	chr6	33382881	A	G	Gln400Arg	missense
1T	PIGV	NM_017837.3	chr1	27124270	G	A	Gly473Ser	missense
3T	PINK1	NM_032409.2	chr1	20974999	C	G	Asp375Glu	missense
2T	PITRM1	NM_001242307.1	chr10	3199129	T	C	Tyr492Cys	missense
4T	PLCB1	NM_015192.2	chr20	8665686	C	A	His324Asn	missense
5T	PLCB3	NM_001184883.1	chr11	64031516	C	T	Pro795Ser	missense
2T	PLCH1	NM_014996.2	chr3	155301409	T	A	Gln178Leu	missense
2T	PLEKHA5	NM_019012.5	chr12	19496356	A	G	Thr781Ala	missense
4T	PLK5	NM_001243079.1	chr19	1531871	A	C	Thr235Pro	missense
5T	PLXNA2	NM_025179.3	chr1	208202263	T	A	Thr1784Ser	missense
5T	PLXNA4	NM_020911.1	chr7	131844338	C	A	Lys1518Asn	missense
5T	PLXNA4	NM_020911.1	chr7	132174151	A	T	Val424Asp	missense
2T	POP1	NM_001145860.1	chr8	99158829	G	T	Gly543Val	missense
2T	POTEF	NM_001099771.2	chr2	130832673	T	A	Tyr791Phe	missense
5T	POTEJ	XM_929706.4	chr2	131415128	T	C	Met932Thr	missense
1T	POTEM	NM_001145442.1	chr14	20020056	C	G	Lys55Asn	missense
1T	POTEM	NM_001145442.1	chr14	20020055	T	A	Thr56Ser	missense
2T	PPEF2	NM_006239.2	chr4	76805876	T	A	Asn206Ile	missense
3T	PPP1R3A	NM_002711.3	chr7	113518151	G	A	Thr999Ile	missense
3T	PPP1R9A	NM_017650.2	chr7	94827714	T	G	Ile603Arg	missense
2T	PRAMEF15	NM_001098376.2	chr1	13645793	A	T	Gln108Leu	missense
5T	PRAMEF22	NM_001100631.1	chr1	13035603	G	A	Gly21Arg	missense
2T	PRAMEF9	NM_001010890.1	chr1	13424996	A	T	Gln108Leu	missense
2T	PRDM16	NM_199454.2	chr1	3322125	G	C	Ala367Pro	missense
5T	PREP	NM_002726.4	chr6	105730443	T	A	Asn522Tyr	missense
4T	PREX2	NM_024870.2	chr8	69032488	A	T	Thr1188Ser	missense
1T	PRKCSH	NM_001001329.1	chr19	11552104	A	T	Thr134Ser	missense
4T	PRSS42	NM_182702.1	chr3	46874617	C	A	Ala151Ser	missense
5T	PSG3	NM_021016.3	chr19	43233470	G	T	Leu350Ile	missense
5T	PTGFRN	NM_020440.2	chr1	117503877	A	T	Gln409Leu	missense

Sample	Gene	Gene ID	Chromosome	Position	Reference Base	Mutation Base	Convert Type	Mutation Type
3T	PTN	NM_002825.5	chr7	136936048	T	A	His127Leu	missense
5T	PTPN11	NM_002834.3	chr12	112926909	A	T	Gln510Leu	missense
3T	PTPN13	NM_080685.2	chr4	87656828	G	C	Glu745Gln	missense
5T	PTPN4	NM_002830.3	chr2	120712820	A	T	Gln634Leu	missense
5T	PTPRK	NM_001135648.1	chr6	128410916	T	A	Asn462Tyr	missense
5T	PTX3	NM_002852.3	chr3	157160157	T	A	Cys179Ser	missense
1T	RABGGTA	NM_182836.2	chr14	24737126	C	A	Asp396Tyr	missense
2T	RAD21L1	NM_001136566.2	chr20	1223489	G	A	Met361Ile	missense
5T	RAD54L2	NM_015106.2	chr3	51697432	A	T	Lys1467Ile	missense
2T	RANBP2	NM_006267.4	chr2	109352564	A	G	Tyr214Cys	missense
3T	RASAL2	NM_170692.2	chr1	178425938	A	T	Gln765Leu	missense
3T	RASAL3	NM_022904.1	chr19	15574992	G	T	Gln60Lys	missense
1T	RASSF7	NM_001143993.1	chr11	562159	G	T	Ala69Ser	missense
1T	RBM45	NM_152945.2	chr2	178988855	A	G	Gln357Arg	missense
3T	RDH16	NM_003708.3	chr12	57345829	G	T	Pro313Gln	missense
1T	REPS1	NM_031922.3	chr6	139265733	A	G	Ser219Pro	missense
5T	RETSAT	NM_017750.3	chr2	85570849	C	T	Gly536Arg	missense
5T	RETSAT	NM_017750.3	chr2	85570857	G	A	Ala533Val	missense
2T	RFTN2	NM_144629.2	chr2	198460743	A	T	Met402Lys	missense
4T	RGS7	NM_002924.4	chr1	240977008	T	A	Gln289Leu	missense
2T	RGS5TP	NM_001029875.1	chr5	63905039	A	C	Lys245Thr	missense
2T	RHBDL2	NM_017821.3	chr1	39361672	A	T	Leu193Gln	missense
1T	RHOBTB1	NM_014836.4	chr10	62671122	C	G	Arg60Pro	missense
3T	RLTPR	NM_001013838.1	chr16	67688482	A	T	Ser1157Cys	missense
5T	RNF38	NM_194330.2	chr9	36351176	G	T	Pro317His	missense
2T	ROBO1	NM_002941.3	chr3	78688970	G	T	His987Gln	missense
5T	RPGRIP1L	NM_001127897.1	chr16	53726139	T	A	Glu123Val	missense
2T	RPS3A	NM_001006.4	chr4	152024228	A	T	Lys187Ile	missense
2T	RREB1	NM_001168344.1	chr6	7231841	T	G	Val1170Gly	missense
2T	RSBN1L	NM_198467.2	chr7	77378954	A	T	Lys306Met	missense
2T	RTN1	NM_021136.2	chr14	60213180	A	T	Ser87Arg	missense
2T	RTTN	NM_173630.3	chr18	67671398	T	A	Asn2224Tyr	missense
4T	RXFP1	NM_001253728.1	chr4	159568318	T	A	Ile541Asn	missense
5T	RYR2	NM_001035.2	chr1	237754112	T	A	Leu1327His	missense
5T	RYR2	NM_001035.2	chr1	237993845	T	A	Cys4891Ser	missense
4T	RYR3	NM_001036.3	chr15	33831643	G	T	Val176Leu	missense
4T	SACS	NM_014363.4	chr13	23915220	T	A	His932Leu	missense
4T	SACS	NM_014363.4	chr13	23929882	T	A	Gln290Leu	missense
5T	SBF2	NM_030962.3	chr11	10050026	T	A	Ser198Cys	missense
2T	SCIN	NM_001112706.2	chr7	12617698	G	T	Cys70Phe	missense
5T	SCN2A	NM_001040142.1	chr2	166237637	A	T	Gln1494Leu	missense
5T	SCN7A	NM_002976.3	chr2	167327193	A	T	Leu199Gln	missense

Sample	Gene	Gene ID	Chromosome	Position	Reference Base	Mutation Base	Convert Type	Mutation Type
5T	SDF2	NM_006923.3	chr17	26976089	T	C	Asn185Ser	missense
5T	SDF4	NM_016176.3	chr1	1154008	C	A	Asp248Tyr	missense
1T	SDK2	NM_001144952.1	chr17	71364703	G	T	Asn1670Lys	missense
2T	SEC11A	NM_014300.2	chr15	85224050	T	A	Ile109Phe	missense
5T	SEC16A	NM_014866.1	chr9	139358015	T	A	Gln1516Leu	missense
2T	SEC24C	NM_198597.2	chr10	75526556	G	A	Arg613Lys	missense
4T	SECISBP2L	NM_014701.3	chr15	49309825	C	A	Asp346Tyr	missense
5T	SEMA4B	NM_020210.3	chr15	90771800	C	A	Ser813Arg	missense
5T	SFXN4	NM_213649.1	chr10	120920579	C	A	Trp89Cys	missense
1T	SGSM3	NM_015705.4	chr22	40803271	G	A	Arg436Gln	missense
3T	SIK2	NM_015191.1	chr11	111571713	A	T	Glu194Asp	missense
5T	SIRPG	NM_018556.3	chr20	1617080	A	G	Cys168Arg	missense
5T	SLC18A2	NM_003054.4	chr10	119026512	T	A	Leu363Gln	missense
5T	SLC22A10	NM_001039752.3	chr11	63058037	A	C	Lys134Gln	missense
5T	SLC30A8	NM_001172811.1	chr8	118183282	A	T	Lys231Met	missense
5T	SLC32A1	NM_080552.2	chr20	37356692	A	G	Met330Val	missense
3T	SLC34A2	NM_006424.2	chr4	25673304	A	G	Thr337Ala	missense
4T	SLC43A2	NM_152346.1	chr17	1494756	G	C	Ile246Met	missense
5T	SLC46A3	NM_181785.3	chr13	29286841	T	A	Ser346Cys	missense
5T	SLC4A11	NM_001174089.1	chr20	3209007	A	T	Leu819Gln	missense
2T	SLC50A1	NM_018845.3	chr1	155109402	C	T	Ala86Val	missense
5T	SLC7A8	NM_012244.3	chr14	23596420	T	A	Lys525Met	missense
1T	SLC8A1	NM_001112802.1	chr2	40655622	T	A	Asp600Val	missense
2T	SLC9A3	NM_004174.2	chr5	482779	G	T	Leu414Met	missense
2T	SLC9A8	NM_001260491.1	chr20	48467369	A	T	Asn202Ile	missense
5T	SLFN11	NM_001104587.1	chr17	33690025	C	A	Asp268Tyr	missense
5T	SLIT1	NM_003061.2	chr10	98763901	A	T	Asp1263Glu	missense
5T	SLITRK5	NM_015567.1	chr13	88329660	G	C	Val673Leu	missense
2T	SMARCAL1	NM_014140.3	chr2	217279522	A	T	Gln32Leu	missense
5T	SNRNP48	NM_152551.3	chr6	7593972	A	T	Glu54Asp	missense
2T	SOBP	NM_018013.3	chr6	107811822	T	A	Met1Lys	missense
5T	SOGA3	NM_001012279.2	chr6	127797342	A	T	Val610Glu	missense
5T	SON	NM_138927.1	chr21	34924575	A	T	Tyr1013Phe	missense
5T	SPATA12	NM_181727.1	chr3	57107798	T	A	Ser26Thr	missense
5T	SPATA12	NM_181727.1	chr3	57107799	C	A	Ser26Tyr	missense
4T	SPDL1	NM_017785.4	chr5	169028478	A	T	Ile507Leu	missense
5T	SPSB1	NM_025106.3	chr1	9416324	A	T	His125Leu	missense
2T	SPTBN2	NM_006946.2	chr11	66472936	T	C	Tyr604Cys	missense
5T	SPTBN5	NM_016642.2	chr15	42145559	T	A	His3321Leu	missense
5T	SSH2	NM_033389.2	chr17	27959340	T	A	Ser931Cys	missense
2T	SSH3	NM_017857.3	chr11	67075102	G	C	Ala229Pro	missense
2T	ST18	NM_014682.2	chr8	53084909	T	G	His171Pro	missense

Sample	Gene	Gene ID	Chromosome	Position	Reference Base	Mutation Base	Convert Type	Mutation Type
5T	ST6GALNAC3	NM_152996.2	chr1	76877851	G	T	Met124Ile	missense
4T	STAB2	NM_017564.9	chr12	104030933	T	A	Cys210Ser	missense
2T	STAG1	NM_005862.2	chr3	136183783	T	C	His418Arg	missense
4T	STARD7	NM_020151.3	chr2	96861148	C	T	Val144Met	missense
5T	STARD8	NM_014725.4	chrX	67942469	C	T	Thr847Met	missense
5T	SUSD5	NM_015551.1	chr3	33194514	A	T	Leu537Gln	missense
2T	SVEP1	NM_153366.3	chr9	113220792	A	T	Ser1179Thr	missense
2T	SYT16	NM_031914.2	chr14	62547876	G	T	Ala440Ser	missense
2T	SYTL2	NM_206927.2	chr11	85435898	T	A	Leu534Phe	missense
5T	TAS1R2	NM_152232.2	chr1	19183923	T	A	Ser129Cys	missense
5T	TENM2	NM_001122679.1	chr5	166802413	G	C	Ser146Thr	missense
4T	TENM4	NM_001098816.2	chr11	78413375	A	T	Val1428Glu	missense
4T	TENM4	NM_001098816.2	chr11	78567209	A	T	Phe424Ile	missense
5T	TENM4	NM_001098816.2	chr11	78381220	T	A	Tyr2057Phe	missense
1T	TG	NM_003235.4	chr8	134034377	T	A	Phe2340Ile	missense
5T	TGM6	NM_198994.2	chr20	2411598	C	A	Pro631His	missense
4T	TIMELESS	NM_003920.3	chr12	56822179	G	T	Phe473Leu	missense
4T	TIMM44	NM_006351.3	chr19	7998770	T	A	Glu221Val	missense
5T	TIMP4	NM_003256.3	chr3	12198344	T	A	Ser110Cys	missense
5T	TLE4	NM_007005.3	chr9	82267689	A	T	His191Leu	missense
5T	TMC2	NM_080751.2	chr20	2597897	T	A	Leu707His	missense
2T	TMC5	NM_001105248.1	chr16	19485464	T	A	Ser652Arg	missense
3T	TMCC1	NM_001128224.2	chr3	129389911	T	C	Asn144Ser	missense
3T	TMEM178A	NM_001167959.1	chr2	39944343	C	G	Ser100Arg	missense
5T	TMEM189-UBE2V1	NM_199203.2	chr20	48713324	T	C	Glu242Gly	missense
4T	TMEM44T	NM_138788.3	chr11	129724666	T	A	Leu114Met	missense
5T	TNFAIP8L2-SCNM1	NM_001204848.1	chr1	151140654	C	A	Pro110Thr	missense
4T	TNN	NM_022093.1	chr1	175052982	A	T	Lys382Met	missense
2T	TNXB	NM_019105.6	chr6	32036793	A	T	Val1903Glu	missense
4T	TPH2	NM_173353.3	chr12	72425096	A	T	Gln408Leu	missense
4T	TPTE	NM_199259.2	chr21	10907008	A	T	Leu500Gln	missense
4T	Trbv27	TRBV27	chr7	142423256	T	A	Leu14Gln	missense
2T	TRIM64C	NM_001206631.1	chr11	49075387	A	T	Leu411Gln	missense
3T	TRIO	NM_007118.2	chr5	14378205	A	T	Gln1139Leu	missense
4T	TRIP11	NM_004239.3	chr14	92469864	T	A	Asn1486Tyr	missense
4T	TRPA1	NM_007332.2	chr8	72936050	G	C	Arg1050Gly	missense
3T	TSPY1	NM_003308.3	chrY	9304872	A	G	Glu88Gly	missense
5T	TSSK1B	NM_032028.3	chr5	112770482	C	T	Gly19Arg	missense
5T	TTC27	NM_017735.4	chr2	32903924	A	T	Thr352Ser	missense
1T	TTF1	NM_001205296.1	chr9	135254455	T	A	Thr304Ser	missense

Sample	Gene	Gene ID	Chromosome	Position	Reference Base	Mutation Base	Convert Type	Mutation Type
5T	TTK	NM_003318.4	chr6	80746278	A	T	Met671Leu	missense
5T	TTLL5	NM_015072.4	chr14	76198720	A	T	Lys361Met	missense
5T	TYR	NM_000372.4	chr11	88911653	T	A	Trp178Arg	missense
5T	TYRP1	NM_000550.2	chr9	12704699	A	T	Asn419Tyr	missense
5T	UBQLN1	NM_013438.4	chr9	86297929	T	A	Thr129Ser	missense
5T	UFL1	NM_015323.4	chr6	96971102	G	A	Gly53Glu	missense
2T	UNC5C	NM_003728.3	chr4	96166143	T	C	Thr310Ala	missense
1T	UNC79	NM_020818.3	chr14	94004491	A	G	Thr250Ala	missense
2T	UNC79	NM_020818.3	chr14	94053131	A	T	Asp793Val	missense
5T	UPF2	NM_080599.2	chr10	12071453	T	A	Ser146Cys	missense
2T	USH2A	NM_206933.2	chr1	215844382	T	A	Ile4689Phe	missense
5T	USP17L7	NM_001256869.1	chr8	11990743	T	A	Gln259Leu	missense
2T	UTP14A	NM_006649.3	chrX	129042696	A	G	Asn75Ser	missense
2T	UTP15	NM_032175.2	chr5	72865470	A	T	Ser173Cys	missense
2T	VEPH1	NM_001167915.1	chr3	157177949	A	T	Leu184Met	missense
2T	VIM	NM_003380.3	chr10	17275863	T	A	Val272Glu	missense
4T	VIT	NM_001177971.1	chr2	37035638	C	A	Phe434Leu	missense
2T	VSIG2	NM_014312.3	chr11	124619649	G	C	Arg181Gly	missense
5T	VWA3T	NM_144992.4	chr2	98844729	A	T	Gln695Leu	missense
5T	WBP1L	NM_001083913.1	chr10	104573000	A	C	His335Pro	missense
2T	WDFY1	NM_020830.3	chr2	224759015	T	G	Gln256Pro	missense
4T	WDFY3	NM_014991.4	chr4	85603574	G	A	Pro3259Leu	missense
2T	WDR52	NM_001164496.1	chr3	113128050	T	C	Ile265Val	missense
5T	WDR87	NM_031951.3	chr19	38379074	A	T	Leu1707Gln	missense
5T	WDR87	NM_031951.3	chr19	38385420	T	A	Lys269Met	missense
4T	ZBTB34	NM_001099270.1	chr9	129642162	G	A	Ala158Thr	missense
5T	ZBTB46	NM_025224.3	chr20	62421321	A	T	Trp264Arg	missense
2T	ZBTB9	NM_152735.3	chr6	33424265	A	T	Gln463Leu	missense
2T	ZDBF2	NM_020923.1	chr2	207175371	G	A	Arg2040Gln	missense
5T	ZFP28	NM_020828.1	chr19	57061939	T	A	Met288Lys	missense
5T	ZFP3	NM_153018.2	chr17	4995752	T	A	Phe318Tyr	missense
2T	ZFP57	NM_001109809.2	chr6	29641317	A	T	Tyr191Asn	missense
2T	ZKSCAN8	NM_006298.2	chr6	28121416	A	T	Gln453Leu	missense
2T	ZMIZ2	NM_031449.3	chr7	44805137	A	C	Gln734Pro	missense
5T	ZMYM2	NM_003453.3	chr13	20641079	A	G	Tyr1074Cys	missense
5T	ZNF235	NM_004234.4	chr19	44792338	T	A	His417Leu	missense
2T	ZNF28	NM_006969.3	chr19	53304026	T	A	Asn358Tyr	missense
1T	ZNF286A	NM_020652.2	chr17	15620417	A	G	His460Arg	missense
4T	ZNF292	NM_015021.1	chr6	87966934	T	C	Leu1196Ser	missense
5T	ZNF324B	NM_207395.2	chr19	58966898	A	T	Lys196Met	missense
1T	ZNF430	NM_001172671.1	chr19	21239661	G	T	Val182Leu	missense
5T	ZNF474	NM_207317.1	chr5	121487764	A	T	Ile27Phe	missense

Sample	Gene	Gene ID	Chromosome	Position	Reference Base	Mutation Base	Convert Type	Mutation Type
5T	ZNF493	NM_001076678.2	chr19	21607515	A	T	Tyr685Phe	missense
2T	ZNF518B	NM_053042.2	chr4	10444802	A	T	Trp1051Arg	missense
5T	ZNF600	NM_198457.2	chr19	53268902	A	T	Phe703Ile	missense
4T	ZNF622	NM_033414.2	chr5	16451815	T	G	Lys462Thr	missense
5T	ZNF695	NM_020394.4	chr1	247150948	T	A	His290Leu	missense
2T	ZNF735	NM_001159524.1	chr7	63680489	A	T	Thr354Ser	missense
5T	ZNF804A	NM_194250.1	chr2	185801241	A	T	Gln373Leu	missense
4T	ZNF85	NM_003429.4	chr19	21132147	A	G	His276Arg	missense
2T	ZNF880	NM_001145434.1	chr19	52888185	T	A	Leu451Gln	missense
5T	ZSCAN20	NM_145238.3	chr1	33956727	A	T	Tyr290Phe	missense
2T	ZSWIM1	NM_080603.4	chr20	44511908	T	C	Leu226Pro	missense
2T	ABCC1	NM_004996.3	chr16	16138308	C	T	Gln271***	nonsense
5T	ADAM18	NM_014237.2	chr8	39468058	C	T	Gln119***	nonsense
2T	ADAMTSL1	NM_001040272.5	chr9	18622291	T	A	Cys175***	nonsense
5T	AP5M1	NM_018229.3	chr14	57747084	A	T	Lys298***	nonsense
5T	ATRN	NM_139321.2	chr20	3559291	A	T	Lys806***	nonsense
1T	C3orf70	NM_001025266.1	chr3	184801113	A	C	Tyr145***	nonsense
2T	CELA2A	NM_033440.2	chr1	15789352	A	T	Lys118***	nonsense
5T	CHRM5	NM_012125.3	chr15	34356366	T	A	Leu483***	nonsense
5T	COL12A1	NM_004370.5	chr6	75898124	G	T	Cys317***	nonsense
5T	CSMD3	NM_052900.2	chr8	113504739	T	A	Arg1649***	nonsense
2T	DHX8	NM_004941.1	chr17	41570206	A	T	Arg221***	nonsense
2T	ELP3	NM_018091.5	chr8	27967944	C	T	Arg151***	nonsense
2T	EPHB6	NM_004445.3	chr7	142562095	G	A	Trp179***	nonsense
5T	ERLIN2	NM_007175.6	chr8	37611045	A	T	Lys273***	nonsense
4T	ESRRG	NM_001134285.2	chr1	216850446	G	T	Cys125***	nonsense
5T	FBN2	NM_001999.3	chr5	127645766	A	T	Cys1703***	nonsense
2T	HIVEP2	NM_006734.3	chr6	143092731	T	A	Lys1049***	nonsense
4T	IGHV3-38	IGHV3-38	chr14	106866489	T	A	Arg89***	nonsense
2T	KCNT1	NM_020822.2	chr9	138671278	A	T	Lys935***	nonsense
5T	KRTAP19-8	NM_001099219.1	chr21	32410676	A	T	Tyr29***	nonsense
5T	MAST4	NM_001164664.1	chr5	66400389	A	T	Lys448***	nonsense
5T	MCTP2	NM_018349.3	chr15	94913334	A	T	Lys503***	nonsense
5T	MMP16	NM_005941.4	chr8	89068496	A	T	Tyr411***	nonsense
2T	NES	NM_006617.1	chr1	156642475	C	T	Trp502***	nonsense
2T	NUCKS1	NM_022731.4	chr1	205688697	T	A	Arg164***	nonsense
5T	OR1L8	NM_001004454.1	chr9	125330100	A	T	Tyr219***	nonsense
2T	OR8I2	NM_001003750.1	chr11	55861230	T	A	Tyr149***	nonsense
5T	POU3F4	NM_000307.3	chrX	82764092	A	T	Lys254***	nonsense
4T	PTPN13	NM_080685.2	chr4	87694021	T	A	Tyr1758***	nonsense
5T	RAB6A	NM_198896.1	chr11	73427381	T	A	Lys106***	nonsense
2T	RALGDS	NM_001042368.1	chr9	135975733	T	A	Lys776***	nonsense

Sample	Gene	Gene ID	Chromosome	Position	Reference Base	Mutation Base	Convert Type	Mutation Type
5T	SLC4A10	NM_001178016.1	chr2	162719546	C	G	Ser258***	nonsense
5T	SLC9C1	NM_183061.1	chr3	111999579	A	T	Leu47***	nonsense
5T	SOGA2	NM_015210.3	chr18	8720456	A	T	Lys107***	nonsense
5T	SPICE1	NM_144718.3	chr3	113186991	T	A	Lys384***	nonsense
5T	SPTBN1	NM_003128.2	chr2	54880905	A	T	Lys1913***	nonsense
5T	STAG2	NM_001042749.1	chrX	123171407	A	T	Lys107***	nonsense
4T	TAS2R43	NM_176884.2	chr12	11244027	T	A	Arg268***	nonsense
2T	TEX10	NM_017746.3	chr9	103090102	T	A	Lys590***	nonsense
5T	TFAP2D	NM_172238.3	chr6	50696948	T	A	Leu269***	nonsense
5T	TRIOBP	NM_001039141.2	chr22	38119848	A	T	Arg429***	nonsense
5T	USH2A	NM_206933.2	chr1	216462732	T	A	Lys621***	nonsense
5T	WDFY4	NM_020945.1	chr10	50081705	T	A	Leu2304***	nonsense

Supplementary Table 3. The list of small insertions and deletions discovered in five cases revealed by whole-exome sequencing

Sample	Gene	Gene ID	Chromosome	Position	Reference Base	Mutation Base	Amino Acid (protein)	Mutation Type
5T	ALB	NM_000477.5	chr4	74286008	GC	G	fs	deletion
2T	CCDC9	NM_015603.2	chr19	47774985	TGTGTGCGC GCGCGCGC	T	fs	deletion
2T	CCL27	NM_006664.2	chr9	34662570	GGTCTGGG CTC	G	fs	deletion
1T	CHD6	NM_032221.3	chr20	40050637	CCGTA	C	fs	deletion
5T	COL15A1	NM_001855.3	chr9	101818639	CA	C	fs	deletion
5T	CORO7-PAM16	NM_001201479.1	chr16	4407235	AG	A	fs	deletion
2T	CRIP3	NM_206922.2	chr6	43274045	TTCTCAGC	T	fs	deletion
2T	DACT1	NM_001079520.1	chr14	59113913	TA	T	fs	deletion
2T	DCAF8L1	NM_001017930.1	chrX	27999273	AG	A	fs	deletion
4T	DNAH5	NM_001369.2	chr5	13708414	AT	A	fs	deletion
5T	DNAI1	NM_012144.2	chr9	34514396	CT	C	fs	deletion
2T	EPN2	NM_148921.3	chr17	19186904	GC	G	fs	deletion
2T	FAT3	NM_001008781.2	chr11	92088511	TC	T	fs	deletion
5T	FREM1	NM_144966.5	chr9	14842494	TA	T	fs	deletion
2T	GGT1	NM_013430.2	chr22	25017036	CG	C	fs	deletion

Sample	Gene	Gene ID	Chromosome	Position	Reference Base	Mutation Base	Amino Acid (protein)	Mutation Type
5T	GIGYF2	NM_001103147.1	chr2	233655839	CA	C	fs	deletion
1T	GTF3C5	NM_001122823.1	chr9	135933277	TGAGGAGG AGGAGGAA	T	in-frame	deletion
2T	IRAK1BP1	NM_001010844.1	chr6	79608128	AATAT	A	fs	deletion
1T	KRTCAP3	NM_001168364.1	chr2	27666035	ACGGTGGC	A	fs	deletion
1T	LTN1	NM_015565.2	chr21	30303628	TC	T	fs	deletion
2T	MAML2	NM_032427.1	chr11	95825374	TTGCTGCTG C	T	in-frame	deletion
2T	MCM8	NM_032485.4	chr20	5975124	GAC	G	fs	deletion
2T	MUC16	NM_024690.2	chr19	9088927	TG	T	fs	deletion
5T	PDCD4	NM_014456.4	chr10	112657916	GT	G	fs	deletion
2T	PHKB	NM_000293.2	chr16	47621614	TG	T	fs	deletion
2T	PIK3R1	NM_181523.2	chr5	67593261	TTG	T	fs	deletion
4T	POU2F1	NM_002697.3	chr1	167385324	TA	T	fs	deletion
2T	PTPRM	NM_002845.3	chr18	7949261	AT	A	fs	deletion
5T	RYR3	NM_001036.3	chr15	34023716	GTA	G	fs	deletion
5T	TARBP1	NM_005646.3	chr1	234601454	ACT	A	fs	deletion
2T	TSR1	NM_018128.4	chr17	2239785	TC	T	fs	deletion
1T	UPK2	NM_006760.3	chr11	118828874	GC	G	fs	deletion
5T	ZEB1	NM_001174093.1	chr10	31816340	AAC	A	fs	deletion
1T	ZNF148	NM_021964.2	chr3	124951171	CTT	C	fs	deletion
1T	APOD	NM_001647.3	chr3	195295708	G	GGGGGT	fs	insertion
3T	ATF7IP	NM_018179.3	chr12	14631383	C	CCA	fs	insertion
1T	CDC27	NM_001114091.1	chr17	45266575	G	GCACCCC	in-frame	insertion
2T	CDCP2	NM_201546.2	chr1	54605318	T	TG	fs	insertion
1T	CDH12	NM_004061.3	chr5	21751718	A	AAAGAG TGTGTGT	in-frame	insertion
3T	CELSR1	NM_014246.1	chr22	46773019	G	GC	fs	insertion
2T	CKAP2	NM_001098525.1	chr13	53049033	G	GA	fs	insertion

Sample	Gene	Gene ID	Chromosome	Position	Reference Base	Mutation Base	Amino Acid (protein)	Mutation Type
3T	DARS	NM_001349.2	chr2	136664812	C	CTTTTTT TTTT	fs	insertion
2T	FAM169B	NM_182562.2	chr15	98980910	T	TG	fs	insertion
4T	FAM171B	NM_177454.3	chr2	187559029	A	ACAGCA G	in-frame	insertion
1T	FBXO9	NM_033481.3	chr6	52962702	A	ATGTG	fs	insertion
2T	GGT5	NM_004121.2	chr22	24640638	G	GCCAGCC CCAGCC	in-frame	insertion
2T	GPM6A	NM_201591.2	chr4	176733449	G	GTGTTT	fs	insertion
1T	HRH2	NM_001131055.1	chr5	175110094	C	CA	fs	insertion
5T	HTR3D	NM_182537.2	chr3	183756836	A	AT	fs	insertion
4T	IAH1	NM_001039613.1	chr2	9621453	G	GCT	fs	insertion
5T	KIAA2026	NM_001017969.2	chr9	5919543	A	AACACA CACACAC ACAC	fs	insertion
3T	KIFC1	NM_002263.3	chr6	33377482	C	CTCTGTG TGTG	fs	insertion
4T	KRT33A	NM_004138.3	chr17	39506946	C	CA	fs	insertion
2T	LRSAM1	NM_138361.5	chr9	130248020	A	AT	fs	insertion
2T	MASP1	NM_001879.5	chr3	187009803	C	CTG	fs	insertion
3T	MCM8	NM_032485.4	chr20	5975124	G	GACAC	fs	insertion
5T	NCOR2	NM_001077261.3	chr12	124824721	C	CGCCGCT GCT	in-frame	insertion
1T	NSUN6	NM_182543.2	chr10	18834797	T	TAAAAA	fs	insertion
5T	OVCH2	NM_198185.3	chr11	7716915	A	AAG	fs	insertion
3T	PKHD1	NM_138694.3	chr6	51656083	A	AT	fs	insertion
2T	PLAA	NM_001031689.2	chr9	26905838	C	CA	fs	insertion
5T	PPP4R4	NM_058237.1	chr14	94745083	G	GTT	fs	insertion
4T	PRKACG	NM_002732.3	chr9	71629038	C	CGCGGC GGCG	in-frame	insertion
4T	RND3	NM_001254738.1	chr2	151343978	G	GA	fs	insertion
1T	RYR2	NM_001035.2	chr1	237969493	C	CT	fs	insertion
1T	SOX9	NM_000346.3	chr17	70117767	C	CAGGT	fs	insertion

Sample	Gene	Gene ID	Chromosome	Position	Reference Base	Mutation Base	Amino Acid (protein)	Mutation Type
3T	SPINT1	NM_181642.2	chr15	41149322	T	TCTCAGC TAAG	fs	insertion
2T	TAOK3	NM_016281.3	chr12	118588761	C	CT	fs	insertion
3T	TMEM180	NM_024789.3	chr10	104235799	G	GTTT	in-frame	insertion
5T	TMEM247	NM_001145051.2	chr2	46707812	G	GGGAGC A	in-frame	insertion
2T	TOB1	NM_001243877.1	chr17	48941417	C	AAAAA	fs	insertion
4T	ZBTB38	NM_001080412.2	chr3	141164925	T	TA	fs	insertion
3T	ZDHHC11	NM_024786.2	chr5	796029	T	TGG	fs	insertion
2T	ZFHX3	NM_001164766.1	chr16	72820938	C	CTT	fs	insertion
1T	HES1	NM_005524.3	chr3	193854148	T	TAAA	in-frame	insertion
3T	LOC100505549	NM_001242804.1	chr18	55335786	T	AAAA	in-frame	insertion
2T	RIN2	NM_001242581.1	chr20	19867256	C	CTTT	in-frame	insertion
5T	FGG	NM_000509.4	chr4	155525429	ATAT	A	in-frame	insertion
2T	LY6G5C	NM_025262.3	chr6	31648129	CAGG	C	in-frame	insertion

Abbreviation; fs, frame shift.

Supplementary Table 4. GO analysis of the biological processes of the affected SNVs

GO Term	P-Value	FDR	Enrichment	(-log2P)
cell adhesion	1.16757E-06	0.002100461	2.458293411	19.70805755
axon guidance	1.42635E-05	0.012830009	2.594562447	16.09731351
regulation of pH	0.000248421	0.130866661	8.154339119	11.97492519
vascular endothelial growth factor signaling pathway	0.000363721	0.130866661	16.30867824	11.42488186
cellular response to caffeine	0.000363721	0.130866661	16.30867824	11.42488186
regulation of cardiac muscle contraction by regulation of the release of sequestered calcium ion	0.000460107	0.13795543	9.513395639	11.08574282
glucose homeostasis	0.000644791	0.165711174	3.805358255	10.59888176
calcium ion homeostasis	0.000738193	0.166001075	8.394172622	10.40371498
regulation of cell migration	0.001188491	0.23756623	4.228175839	9.716652794
ATP catabolic process	0.00178769	0.321098262	2.748314296	9.127687939
regulation of heart rate	0.001963358	0.321098262	6.486406117	8.992461091
axonogenesis	0.003127977	0.451134888	3.251413699	8.320554394
actin filament capping	0.005020634	0.451134888	6.715338098	7.637914595
homophilic cell adhesion	0.006149394	0.451134888	2.446301736	7.345339997

GO Term	P-Value	FDR	Enrichment	(-log2P)
Purkinje myocyte to ventricular cardiac muscle cell signaling	0.006603405	0.451134888	28.54018692	7.242574135
smoothened signaling pathway involved in spinal cord motor neuron cell fate specification	0.006603405	0.451134888	28.54018692	7.242574135
smoothened signaling pathway involved in ventral spinal cord interneuron specification	0.006603405	0.451134888	28.54018692	7.242574135
spinal cord ventral commissure morphogenesis	0.006603405	0.451134888	28.54018692	7.242574135
trigeminal nerve morphogenesis	0.006603405	0.451134888	28.54018692	7.242574135
calcium-mediated signaling using intracellular calcium source	0.006844968	0.451134888	9.513395639	7.190740455
sensory perception of light stimulus	0.006844968	0.451134888	9.513395639	7.190740455
positive regulation vascular endothelial growth factor production	0.007036607	0.451134888	6.008460403	7.150904364
hemopoiesis	0.008355894	0.451134888	3.275103417	6.902990124
calcium ion transport into cytosol	0.008674667	0.451134888	8.562056075	6.848975877
regulation of neuron projection development	0.008674667	0.451134888	8.562056075	6.848975877
melanin biosynthetic process	0.008674667	0.451134888	8.562056075	6.848975877

Supplementary Table 5. Pathway analysis of the affected SNVs

Pathway Term	P-Value	FDR	Enrichment	(-log2P)
ECM-receptor interaction	0.000190609	0.036215772	3.875200829	12.35709366
Focal adhesion	0.002488706	0.186761846	2.321585903	8.650388271
Amoebiasis	0.003052019	0.186761846	2.87564506	8.356020201
Protein digestion and absorption	0.004895455	0.186761846	3.049926579	7.674341431
Axon guidance	0.004914785	0.186761846	2.553426903	7.668655858
ABC transporters	0.008395866	0.241392317	3.74309171	6.89610514
Arrhythmogenic right ventricular cardiomyopathy (ARVC)	0.008893401	0.241392317	2.96749613	6.813049025
Calcium signaling pathway	0.012899819	0.306370695	2.077247291	6.276505398
Hypertrophic cardiomyopathy (HCM)	0.015999568	0.337768668	2.645719442	5.965823194
Legionellosis	0.021063143	0.400199715	2.994473368	5.569135467
Dilated cardiomyopathy	0.02382917	0.411594748	2.439941263	5.391127513
Small cell lung cancer	0.050227398	0.79526713	2.208567522	4.31538166
Vitamin digestion and absorption	0.068923601	0.913955661	3.431167401	3.858858116
Pathways in cancer	0.073122947	0.913955661	1.506366176	3.773531967
Valine, leucine and isoleucine biosynthesis	0.07526237	0.913955661	4.990788947	3.731927474
Olfactory transduction	0.081514376	0.913955661	1.437496567	3.616801673
Glycosaminoglycan biosynthesis - heparan sulfate	0.08177498	0.913955661	3.167231447	3.612196684
Serotonergic synapse	0.092411207	0.975451632	1.799956669	3.435788363
Folate biosynthesis	0.108042514	0.99376834	3.921334172	3.210328983
Endocrine and other factor-regulated calcium reabsorption	0.117699018	0.99376834	2.240762384	3.086825811

Pathway Term	P-Value	FDR	Enrichment	(-log2P)
Viral myocarditis	0.126965424	0.99376834	1.960667086	2.97749243
Mineral absorption	0.129904697	0.99376834	2.15288935	2.944474501
Endocytosis	0.139007575	0.99376834	1.494765996	2.846764591
African trypanosomiasis	0.141831378	0.99376834	2.422000518	2.817751356
Cardiac muscle contraction	0.142844089	0.99376834	1.880091727	2.807486761
Prion diseases	0.150157253	0.99376834	2.352800503	2.735453934
Staphylococcus aureus infection	0.155764313	0.99376834	1.996315579	2.682563356
Ribosome biogenesis in eukaryotes	0.15952124	0.99376834	1.805877579	2.648179567
Butirosin and neomycin biosynthesis	0.193894872	0.99376834	5.489867841	2.36665345
Tryptophan metabolism	0.212114102	0.99376834	1.960667086	2.237087556
Tyrosine metabolism	0.212114102	0.99376834	1.960667086	2.237087556
Synaptic vesicle cycle	0.219753834	0.99376834	1.7155837	2.186039757
Tight junction	0.223165602	0.99376834	1.455646776	2.163813421
Carbohydrate digestion and absorption	0.230722754	0.99376834	1.871545855	2.115767806
Valine, leucine and isoleucine degradation	0.230722754	0.99376834	1.871545855	2.115767806
RNA transport	0.250614203	0.99376834	1.363942321	1.996459917
Notch signaling pathway	0.25913148	0.99376834	1.752085481	1.948243805
Long-term depression	0.265617634	0.99376834	1.568533669	1.912577168
Amino sugar and nucleotide sugar metabolism	0.26870103	0.99376834	1.7155837	1.895926241
Vascular smooth muscle contraction	0.269399304	0.99376834	1.407658421	1.892181972
Pertussis	0.297066219	0.99376834	1.483748065	1.751143535
Taste transduction	0.297603459	0.99376834	1.614667012	1.748536798
Galactose metabolism	0.299415155	0.99376834	1.893057876	1.739780851
Synthesis and degradation of ketone bodies	0.301710885	0.99376834	3.049926579	1.728761348
Neuroactive ligand-receptor interaction	0.310550981	0.99376834	1.206564361	1.687097971
Cell cycle	0.311798511	0.99376834	1.328193833	1.681314058
O-Glycan biosynthesis	0.312523579	0.99376834	1.829955947	1.677963053
Pancreatic secretion	0.320390185	0.99376834	1.358878179	1.642098144
Melanogenesis	0.320390185	0.99376834	1.358878179	1.642098144
Histidine metabolism	0.325575393	0.99376834	1.77092511	1.618936429
Sulfur relay system	0.326322095	0.99376834	2.744933921	1.61563142
Retrograde endocannabinoid signaling	0.327305499	0.99376834	1.345555843	1.611290256
Basal cell carcinoma	0.336331855	0.99376834	1.497236684	1.572042669
Starch and sucrose metabolism	0.336331855	0.99376834	1.497236684	1.572042669
Hedgehog signaling pathway	0.346005116	0.99376834	1.470500315	1.531134726
Riboflavin metabolism	0.3729622	0.99376834	2.287444934	1.422898676
Toxoplasmosis	0.373820699	0.99376834	1.22907489	1.419581641
Alzheimer's disease	0.382498182	0.99376834	1.193449531	1.386475205
Cholinergic synapse	0.396819506	0.99376834	1.225416929	1.333445152
Colorectal cancer	0.40353375	0.99376834	1.328193833	1.309238756
Salivary secretion	0.416584176	0.99376834	1.233678167	1.263320056
Rheumatoid arthritis	0.432306038	0.99376834	1.206564361	1.209875107
Glycosphingolipid biosynthesis - ganglio series	0.436914649	0.99376834	1.829955947	1.194576616

Pathway Term	P-Value	FDR	Enrichment	(-log2P)
Glycosaminoglycan biosynthesis - keratan sulfate	0.436914649	0.99376834	1.829955947	1.194576616
Parkinson's disease	0.438242665	0.99376834	1.163107594	1.190198152
Epithelial cell signaling in Helicobacter pylori infection	0.459447331	0.99376834	1.211000259	1.122028608
Complement and coagulation cascades	0.468548465	0.99376834	1.193449531	1.093729812
Other glycan degradation	0.475868643	0.99376834	1.614667012	1.071364702
Pantothenate and CoA biosynthesis	0.475868643	0.99376834	1.614667012	1.071364702
Long-term potentiation	0.477578529	0.99376834	1.176400252	1.066190117
Transcriptional misregulation in cancer	0.484186647	0.99376834	1.073437846	1.046364802
Phenylalanine metabolism	0.494318844	0.99376834	1.524963289	1.016486191
Chronic myeloid leukemia	0.504214176	0.99376834	1.128055036	0.987891415
Basal transcription factors	0.508116599	0.99376834	1.193449531	0.976768502
GnRH signaling pathway	0.508691324	0.99376834	1.087102543	0.975137607
Glycosaminoglycan degradation	0.512116822	0.99376834	1.444702064	0.965455145
Dopaminergic synapse	0.518545455	0.99376834	1.055743816	0.947457636
Gastric acid secretion	0.521567353	0.99376834	1.097973568	0.939074527
Regulation of actin cytoskeleton	0.529184967	0.99376834	1.021370761	0.918156018
Type II diabetes mellitus	0.529949751	0.99376834	1.143722467	0.916072522
Antigen processing and presentation	0.530116085	0.99376834	1.083526548	0.91561978
Chagas disease	0.530682414	0.99376834	1.055743816	0.914079355
Osteoclast differentiation	0.531456132	0.99376834	1.039747697	0.911977482
Terpenoid backbone biosynthesis	0.5458479	0.99376834	1.307111391	0.873429093
Cocaine addiction	0.551105173	0.99376834	1.097973568	0.859600426
Malaria	0.561425208	0.99376834	1.076444675	0.832834252
Protein processing in endoplasmic reticulum	0.566606108	0.99376834	0.992144791	0.819581941
Phosphatidylinositol signaling system	0.571508839	0.99376834	1.016642193	0.807152283
Protein export	0.577237377	0.99376834	1.193449531	0.792763376
Proximal tubule bicarbonate reclamation	0.577237377	0.99376834	1.193449531	0.792763376
Mismatch repair	0.577237377	0.99376834	1.193449531	0.792763376
Glycosylphosphatidylinositol(GPI)-anchor biosynthesis	0.606448465	0.99376834	1.097973568	0.721543041
Maturity onset diabetes of the young	0.606448465	0.99376834	1.097973568	0.721543041
Leukocyte transendothelial migration	0.613237142	0.99376834	0.946528938	0.705483016
Arginine and proline metabolism	0.619699378	0.99376834	0.963134709	0.690359574
Hematopoietic cell lineage	0.625439476	0.99376834	0.935772928	0.677057814
Collecting duct acid secretion	0.633632957	0.99376834	1.016642193	0.658280719
Gap junction	0.639951252	0.99376834	0.914977974	0.643966081
MAPK signaling pathway	0.641048888	0.99376834	0.921806167	0.64149371
Homologous recombination	0.646509739	0.99376834	0.980333543	0.629255992
Morphine addiction	0.654058079	0.99376834	0.895087148	0.612509345
Inositol phosphate metabolism	0.655087731	0.99376834	0.899978335	0.610239965
beta-Alanine metabolism	0.658932023	0.99376834	0.946528938	0.601798453

Pathway Term	P-Value	FDR	Enrichment	(-log2P)
Citrate cycle (TCA cycle)	0.670915919	0.99376834	0.914977974	0.57579612
Butanoate metabolism	0.670915919	0.99376834	0.914977974	0.57579612
Fc gamma R-mediated phagocytosis	0.674457028	0.99376834	0.866821238	0.568201568
Glutamatergic synapse	0.674594484	0.99376834	0.871407594	0.567907573
Retinol metabolism	0.70312505	0.99376834	0.81938326	0.508146801
Base excision repair	0.704389891	0.99376834	0.831798158	0.50555389
Pentose and glucuronate interconversions	0.714770163	0.99376834	0.807333506	0.484448683
Selenoamino acid metabolism	0.724784392	0.99376834	0.784266834	0.464376207
Renal cell carcinoma	0.724963026	0.99376834	0.784266834	0.464020676
Pancreatic cancer	0.724963026	0.99376834	0.784266834	0.464020676
Bacterial invasion of epithelial cells	0.724963026	0.99376834	0.784266834	0.464020676
Amphetamine addiction	0.724963026	0.99376834	0.784266834	0.464020676
DNA replication	0.73444554	0.99376834	0.762481645	0.445272577
Fructose and mannose metabolism	0.73444554	0.99376834	0.762481645	0.445272577
Leishmaniasis	0.738748187	0.99376834	0.762481645	0.436845411
Insulin signaling pathway	0.743616942	0.99376834	0.789909042	0.427368455
Glycine, serine and threonine metabolism	0.743766109	0.99376834	0.741874033	0.427079084
Cytokine-cytokine receptor interaction	0.744568875	0.99376834	0.828659297	0.425522786
Adherens junction	0.745414876	0.99376834	0.752036691	0.423884484
Viral carcinogenesis	0.761106155	0.99376834	0.79563302	0.393830409
VEGF signaling pathway	0.764537172	0.99376834	0.722351032	0.387341447
Nicotine addiction	0.769802781	0.99376834	0.68623348	0.377439211
Graft-versus-host disease	0.777877392	0.99376834	0.669496078	0.362385318
Aminoacyl-tRNA biosynthesis	0.777877392	0.99376834	0.669496078	0.362385318
Aldosterone-regulated sodium reabsorption	0.785667571	0.99376834	0.653555695	0.348009082
Peroxisome	0.788068396	0.99376834	0.68623348	0.34360725
Porphyrin and chlorophyll metabolism	0.79318338	0.99376834	0.638356726	0.334273646
Type I diabetes mellitus	0.79318338	0.99376834	0.638356726	0.334273646
Wnt signaling pathway	0.796777853	0.99376834	0.727134813	0.327750547
Phagosome	0.80077259	0.99376834	0.722351032	0.320535502
Fat digestion and absorption	0.81417992	0.99376834	0.596724765	0.296580453
Nucleotide excision repair	0.81417992	0.99376834	0.596724765	0.296580453
Salmonella infection	0.814528766	0.99376834	0.645866805	0.295962445
Lysosome	0.81453814	0.99376834	0.680562129	0.295945842
Progesterone-mediated oocyte maturation	0.819449939	0.99376834	0.638356726	0.287272279
Lysine degradation	0.83303645	0.99376834	0.560190596	0.263548472
N-Glycan biosynthesis	0.83303645	0.99376834	0.560190596	0.263548472
Apoptosis	0.83351267	0.99376834	0.616839083	0.262723966
GABAergic synapse	0.83351267	0.99376834	0.616839083	0.262723966
Glutathione metabolism	0.84452766	0.99376834	0.538222337	0.24378342
Fanconi anemia pathway	0.849971938	0.99376834	0.527871908	0.234512884
Autoimmune thyroid disease	0.849971938	0.99376834	0.527871908	0.234512884
Drug metabolism - other enzymes	0.849971938	0.99376834	0.527871908	0.234512884

Pathway Term	P-Value	FDR	Enrichment	(-log2P)
Amyotrophic lateral sclerosis (ALS)	0.855224763	0.99376834	0.517912061	0.225624468
Cell adhesion molecules (CAMs)	0.859519822	0.99376834	0.619158027	0.218397184
Glycerolipid metabolism	0.860292897	0.99376834	0.508321096	0.217100169
Vibrio cholerae infection	0.860292897	0.99376834	0.508321096	0.217100169
Ubiquitin mediated proteolysis	0.872213832	0.99376834	0.601080421	0.197246226
Natural killer cell mediated cytotoxicity	0.872213832	0.99376834	0.601080421	0.197246226
Acute myeloid leukemia	0.874453211	0.99376834	0.481567355	0.193546902
NOD-like receptor signaling pathway	0.894925066	0.99376834	0.442731278	0.160161207
Cytosolic DNA-sensing pathway	0.894925066	0.99376834	0.442731278	0.160161207
Herpes simplex infection	0.898017373	0.99376834	0.596724765	0.155184739
Arachidonic acid metabolism	0.898598339	0.99376834	0.435703797	0.1542517
T cell receptor signaling pathway	0.901628238	0.99376834	0.508321096	0.149395394
Glycolysis / Gluconeogenesis	0.905562563	0.99376834	0.422297526	0.143113778
Chemokine signaling pathway	0.91063958	0.99376834	0.577880825	0.135047929
Oocyte meiosis	0.912153418	0.99376834	0.490166772	0.132651599
Jak-STAT signaling pathway	0.91750397	0.99376834	0.531277533	0.124213695
Adipocytokine signaling pathway	0.918083506	0.99376834	0.39781651	0.123302712
RIG-I-like receptor signaling pathway	0.923704419	0.99376834	0.386610411	0.114496825
Melanoma	0.923704419	0.99376834	0.386610411	0.114496825
Bile secretion	0.923704419	0.99376834	0.386610411	0.114496825
PPAR signaling pathway	0.923704419	0.99376834	0.386610411	0.114496825
Drug metabolism - cytochrome P450	0.931418107	0.99376834	0.370937016	0.102499166
Purine metabolism	0.937372191	0.99376834	0.496072395	0.0933061
Fc epsilon RI signaling pathway	0.942574118	0.99376834	0.34745999	0.085322027
Spliceosome	0.942876759	0.99376834	0.432273058	0.084858883
Metabolism of xenobiotics by cytochrome P450	0.948373178	0.99376834	0.334748039	0.076473235
TGF-beta signaling pathway	0.951908772	0.99376834	0.326777848	0.071104778
Measles	0.95340409	0.99376834	0.40969163	0.068840281
Glycerophospholipid metabolism	0.953584322	0.99376834	0.322933402	0.068567578
Alcoholism	0.955084789	0.99376834	0.460044791	0.066299278
Tuberculosis	0.955084789	0.99376834	0.460044791	0.066299278
Systemic lupus erythematosus	0.95605155	0.99376834	0.403666753	0.064839684
Ribosome	0.958267389	0.99376834	0.311924309	0.061499822
HTLV-I infection	0.958357518	0.99376834	0.521850555	0.061364138
mRNA surveillance pathway	0.961122819	0.99376834	0.304992658	0.057207294
NF-kappa B signaling pathway	0.962476101	0.99376834	0.30164109	0.055177377
Toll-like receptor signaling pathway	0.974577942	0.99376834	0.269111169	0.037150525
Pyrimidine metabolism	0.974577942	0.99376834	0.269111169	0.037150525
Metabolic pathways	0.985274314	0.99376834	0.666592799	0.021402649
Influenza A	0.985826986	0.99376834	0.315509646	0.020593621
Oxidative phosphorylation	0.98605414	0.99376834	0.230666716	0.020261234
Huntington's disease	0.987441837	0.99376834	0.308419542	0.018232322

Pathway Term	P-Value	FDR	Enrichment	(-log2P)
Neurotrophin signaling pathway	0.990196659	0.99376834	0.212785575	0.014213013
Epstein-Barr virus infection	0.99376834	0.99376834	0.273127753	0.009018515

Supplementary Table 6. Primers used to carry out Sanger sequencing of the frequent mutations

Gene	Forward Primer	Reverse Primer	Amplification Length
DISC1	cagaaagaaatcgaagctctcca	ttatggtttccggtggctct	235
	tcaccttcgaatgtgctcct	cccgggtccaaacatggt	224
USH2A	tgctaccatttctagtgccaac	aggtgcattaaaacggacgt	244
	tgacgggtgcaaacaatcag	ctcttttgtggacaggaccg	223
TENM4	acccggaagctgttgtcata	acgctctatgacaccaccaa	206
	gatggccaccttgcttagc	tgggatggattagtggtggg	241
	tcagatgcacaggatggtct	agatgccctggtttctcgtt	211
UNC79	gaagagcagttgtcacctgc	ggagttgtcagaggcagcta	250
	accaacagcttccggtaaca	tgagggtgatcttttctggca	241
DNAH17	ctcggggaggagctgata	ctttgtgggagcctcttcc	242
	tttcatggacacctctagcctc	tcctttgtcctcagctcctg	257
MUC16	ctgttgttgtggccaaggta	gtgcccactagtgctactact	232
	ccagtccctgtgatgcttct	cagtccacagccctaggaag	59
PTPN13	ggcacaaactaaccatattgaca	accagttcaaagtcttcaaggt	249
	aagggtttactccaataaaaggc	tctaacacttcttgcttacttgga	274
PCLO	tgaggttatcttgggctcca	cttcctacccaagcaccca	246
	ctgttgtggggtgaggga	tcatgtgagaatctcctactacgt	453
PDE1C	tgagtaatttgaatgtcacccca	ggggaagcttcagtggtaga	194
	ggttgtatttaaaggcccatgc	cacctacctgcagctatgga	230

Supplementary Figure 2

Case 1

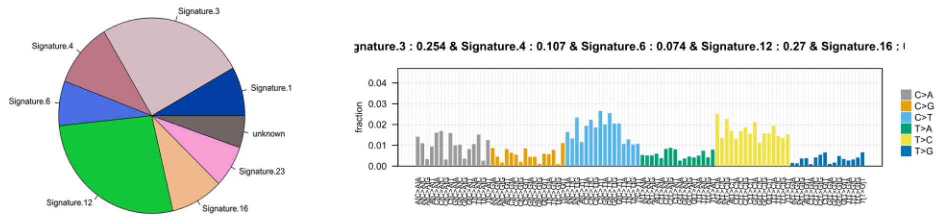

Case 2

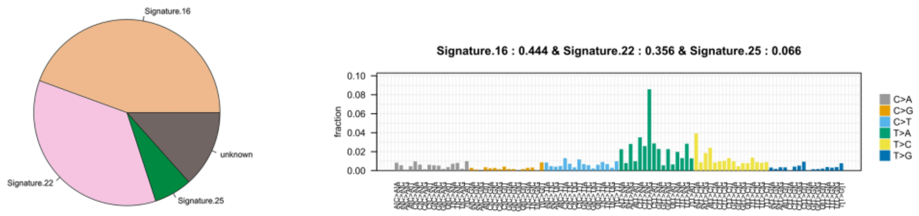

Case 3

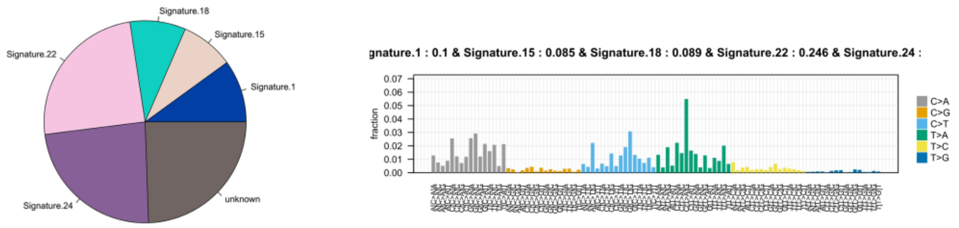

Case 4

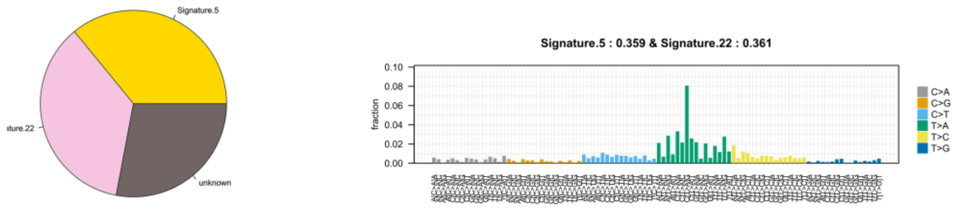

Case 5

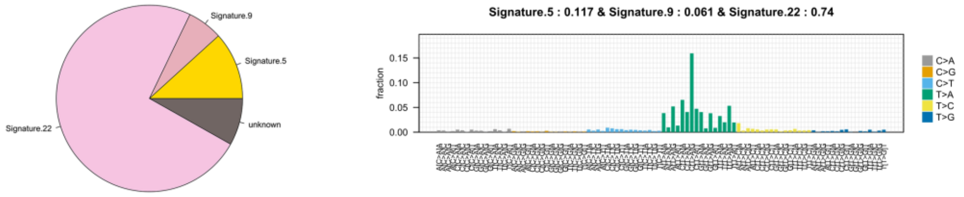

All cases

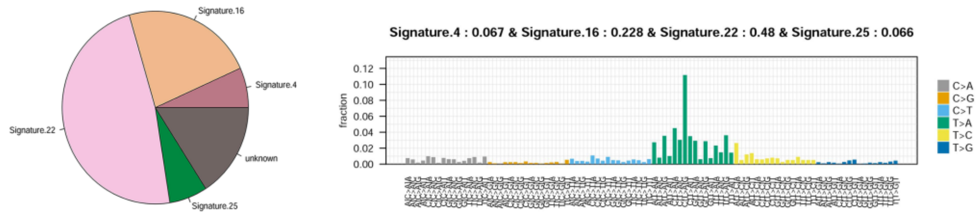

Supplementary Figure 2. The mutational signatures consisting of 96 different mutated trinucleotides identified in each case and all cases together. Signature 22 dominated in 4 out of 5 cases.
